# Dupilumab in Inflammatory Skin Diseases: A Systematic Review

**DOI:** 10.3390/biom13040634

**Published:** 2023-03-31

**Authors:** Henning Olbrich, Christian D. Sadik, Ralf J. Ludwig, Diamant Thaçi, Katharina Boch

**Affiliations:** 1 Department of Dermatology, University of Lübeck, 23566 Lübeck, Germany; 2 Lübeck Institute of Experimental Dermatology, University of Lübeck, 23566 Lübeck, Germany; 3 Institute and Comprehensive Center for Inflammation Medicine, University-Hospital Schleswig-Holstein, 23566 Lübeck, Germany

**Keywords:** dupilumab, atopic dermatitis, review, prurigo nodularis, pruritus, alopecia areata, eczema, contact dermatitis, chronic spontaneous urticaria, bullous pemphigoid

## Abstract

Dupilumab was first approved for the treatment of atopic dermatitis (AD) and blocks the signaling of interleukin (IL)-4 and -13. Several other chronic skin conditions share mechanistic overlaps with AD in their pathophysiology, i.e., are linked to type 2 inflammation. Most recently, dupilumab was approved by the U.S. Food and Drug Administration for prurigo nodularis (PN). Given its relatively good safety profile, effective off-label use of dupilumab has been reported for a multitude of dermatologic diseases and several clinical trials for dermatologic skin conditions are currently ongoing. We conducted a systematic review of applications of dupilumab in dermatology other than AD and PN by searching the databases PubMed/Medline, Scopus, Web of Science and Cochrane Library as well as the clinical trial registry ClinicalTrials.gov. We found several reports for effective treatment of bullous autoimmune diseases, eczema, prurigo, alopecia areata, chronic spontaneous urticaria, Netherton syndrome and a variety of other chronic inflammatory skin diseases.

## 1. Introduction

Dupilumab is a human monoclonal IgG4 antibody directed against the interleukin (IL)-4 receptor alpha chain (IL4Rα) and inhibits signaling of both IL-4 and -13. These cytokines are key mediators of type 2 helper T cell (Th2)-related immune responses that drive atopic and many other inflammatory skin diseases. Th2-responses are associated with eosinophilia, basophil and mast cell recruitment and production of IgE. Dupilumab was first approved by the European Medicines Agency and the U.S. Food and Drug Administration in 2017 for the management of moderate-to-severe atopic dermatitis (AD) in adults, and more recently, in adolescents and children from the age of 6 months. Clinical trials showed long-term improvement of AD-signs and symptoms including pruritus, size and severity of skin lesions and overall quality of life, as well as lower rates of skin infections compared with placebo treatment [1,2,3]. Patient skin samples revealed downregulation of Th2 molecular markers, reduction of cellular infiltrate and an improved skin barrier function [4,5]. Beyond dermatology, dupilumab is also effective and approved for moderate-to-severe asthma, chronic rhinosinusitis with nasal polyposis (CRSwNP) [6] and eosinophilic esophagitis. In addition, efficacy of dupilumab in prurigo nodularis (PN) and other forms of chronic prurigo was previously shown in several reports [7,8,9,10,11,12,13,14,15,16,17,18,19,20,21,22,23,24,25,26,27,28,29,30,31,32]. The phase 3 clinical trials LIBERTY-PN PRIME and PRIME2 showed significantly reduced itch, amelioration of skin lesions, sleep, pain and quality of life compared to placebo treatment in a total of 153 treated patients (NCT04202679, NCT04183335). These findings led to the approval of dupilumab for PN in adults in September of 2022 [33].

**Figure 1:**
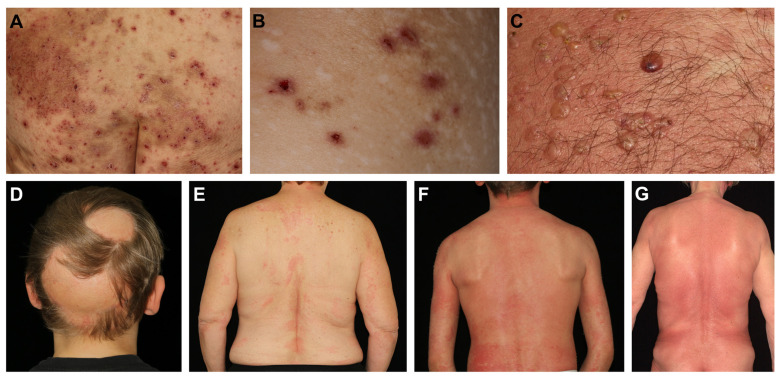
Clinical presentations of chronic inflammatory skin diseases with reports of effective dupilumab treatment. (**A**) Severe nummular eczema with confluent itchy and scaly plaques showing superficial excoriations. (**B**) Chronic prurigo presenting with intensely pruritic nodules that are developed and sustained by pathognomonic itch-scratch cycles. Deep scratching results in visible scars. (**C**) Tense clear or hemorrhagic blisters on reddish and infiltrated skin typically seen in bullous pemphigoid. (**D**) Nonscarring patchy hair loss on the scalp in a patient with alopecia areata. Progression can lead to total hair loss of the head (alopecia totalis, AT) or even the entire body (alopecia universalis, AU). (**E**) Chronic urticaria presenting with recurrent wheals that form and fade in rapid succession and can be accompanied with itch. (**F**) A pediatric patient with Netherton syndrome presenting with severe itchy chronic infiltration, lichenification and papulation of the skin resembling atopic dermatitis. (**G**) Mycosis fungoides in an elderly patient with localized dark patches on the trunk that are highly pruritic.

Adverse events reported most frequently are nasopharyngitis, upper respiratory tract infections, headache, injection-site reactions and conjunctivitis [2]; further, facial and neck erythema was rare but typically attributed to dupilumab [34]. As this indicates relatively high drug safety, dupilumab has been administered off-label in several other dermatologic disease entities known to be associated with Th2-responses, reviewed previously in [35,36,37] (Figure 1). A number of clinical trials are currently ongoing for chronic pruritus of unknown origin, chronic hand eczema, nummular eczema, bullous pemphigoid, alopecia areata, chronic spontaneous, cold or cholinergic urticaria, localized scleroderma, keloids, food allergies and Netherton syndrome.

This systematic review addresses clinical outcomes and potential future use of dupilumab in chronic inflammatory skin conditions.

## 2. Methods

**Figure 2:**
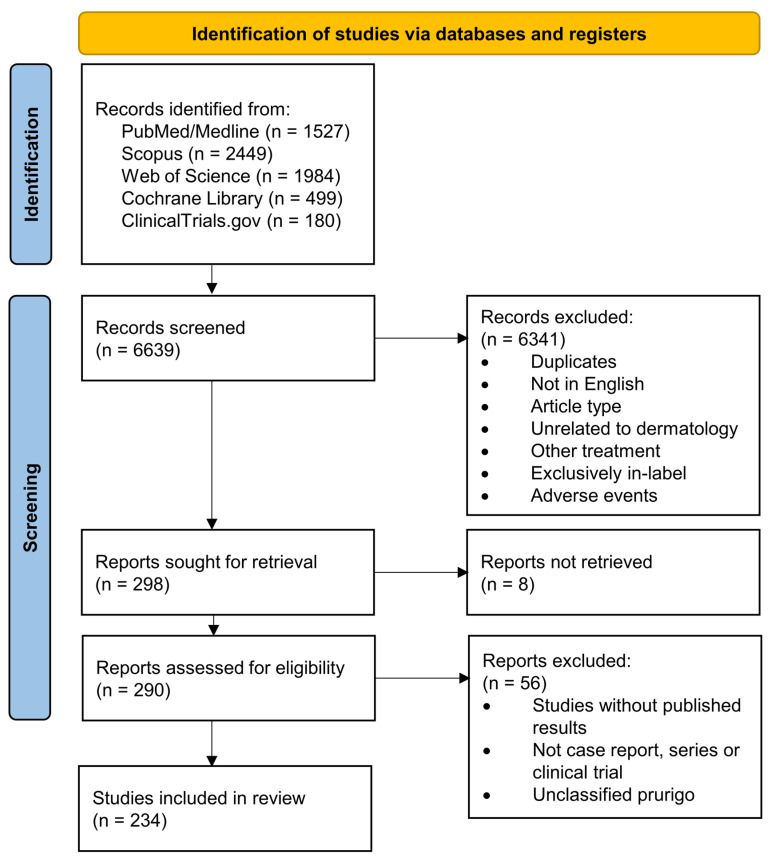
PRISMA flow diagram depicting the different phases of this systematic review.

The databases PubMed/Medline, Scopus, Web of Science and Cochrane Library were queried for the terms (“dermatology” OR “skin” OR “dermatitis”) AND “dupilumab” and reports published before 15 January 2023 were collected. Spelling variants of the query terms were included. Further, the registry ClinicalTrials.gov was queried for “dupilumab” as specialty specifications were frequently not denoted. The search strategy was represented in a flow chart according to the Preferred Reporting Items for Systematic Reviews and Meta-Analyses (PRISMA) [38] (Figure 2).

Duplicate reports were identified automatically by PubMed or ClinicalTrials.gov identifiers (PMID or NCT registry numbers) and excluded. By screening based on titles and abstracts performed by hand, we then excluded duplicate reports not identified previously, articles in languages other than English, all review articles and meeting abstracts. Further, we excluded reports unrelated to the field of dermatology, reports on other drugs than dupilumab, or reports of sole in-label treatment with dupilumab (including treatment for AD or PN in adults), as well as reports of adverse events of in-label dupilumab treatment.

The search was refined by full-text review after excluding articles that could not be retrieved. Here, we excluded reports other than case reports, case series, retrospective clinical studies or randomized controlled trials (RCT), reports that did not clarify clinical responses, and studies that had not yet reported. Further, reports of adult patients with chronic prurigo were excluded if the subtype of prurigo was not elucidated in order to avoid repeated reporting of PN. The levels of evidence were categorized for each report adapting the Oxford Centre for Evidence-Based Medicine (OCEBM) levels of evidence table [39]: Level 5—singular case reports; level 4—case series or uncontrolled retrospective studies; level 3—prospective and interventional studies as well as controlled retrospective studies; level 2—randomized controlled trials and level 1—meta-analyses.

## 3. Results and Discussion

### 3.1. Eczema

Pruritus is often a prime symptom of eczema and hence, eczematous skin lesions frequently show signs of scratching and excoriation (Figure 1A). A variety of eczema entities including atopic hand eczema (HE)/hand dermatitis or nummular eczema (NE) can have clinical and functional overlaps with AD. Still, hand and nummular eczema/dermatitis can develop in non-atopic individuals and without fulfilling diagnostic criteria for AD (e.g., Hanifin’s and Rajka’s criteria), thus, treatment with dupilumab in these conditions is currently off-label, or can be offered to patients with concomitant AD.

NE is characterized by coin-shaped eczematous plaques and was typically associated with skin microbial disbalance; yet, pathogenesis is highly heterogeneous. We identified retrospective case series with a total of 36 patients with NE treated with dupilumab. However, the majority of patients (30/36) had concomitant AD. Significant improvement of skin manifestations measured by the eczema area and severity index (EASI) or affected body surface area (BSA) as well as improvement in pruritus and quality of life was found in all treated patients, including 6 patients without AD. The responses were sustained in all but one patient during a follow-up period of up to 2 years (Table 1) [40,41]. The phase 2 placebo-controlled RCT DUPINUM (NCT04600362) investigating efficacy and safety of dupilumab in adults with NE is currently recruiting. 

Contact dermatitis is frequently associated with the patient’s occupation and can be caused by irritants (irritant-toxic contact dermatitis, ICD) or type-IV allergens (allergic contact dermatitis, ACD). ACD is found more frequently in patients with AD, suggesting a susceptibility to type-IV sensitivities possibly by a defective skin barrier. ACD mechanisms are highly variable and allergen-specific with, e.g., a strong Th1/Th17-polarization found for nickel while fragrances and rubber showed a Th2-bias [42]. Records found for a total of 67 patients with or without concomitant AD that were refractory to topical and systemic steroids as well as other systemic immunosuppressants and subsequently treated with dupilumab showed complete or partial responses in 65/67 patients (Table 1) [43,44,45,46,47,48,49,50,51,52,53,54,55,56,57,58,59]. In two cases, no effects of dupilumab were reported [60,61]. Interestingly, some reports demonstrated clinical remissions under dupilumab when allergen or irritant avoidance was not feasible. Other studies performed patch-testing before and after treatment with dupilumab and found that for most type-IV allergens except for fragrance and balsam of Peru, testing results remained positive despite overall clinical improvement [62]. Thus, larger studies considering allergen specificities and concomitant atopy are needed. Currently, two open-label phase 4 clinical trials evaluating dupilumab in ACD are recruiting (NCT05535738, NCT03935971).

**Table 1:** Eczema.

Disease	Study Type	n	Sex/Age	Presentation	Medical History	Prior Therapies	Concomitant Therapy	Therapy	Response	Reference
Nummular dermatitis	retrospective study (4)	30	38.2 (mean, 19–78 range), females: 12/30	nummular eczema associated with AD (30/30)	AD (30/30)	topical (18/30) and systemic (28/30) steroids, topical calcineurin inhibitors (8/30), ciclosporin (22/30)		Dupilumab 600 mg s.c., then 300 mg q2w	Improvement in skin manifestations (EASI -32.87 mean), itch (NRS -7.17 mean) and quality of life (DLQI -14.57 mean) at 16 wk	[40]
Nummular dermatitis	case series (4)	6	73.5 (median, 45–90 range), females: 1/6	nummular eczema without AD	no AD (0/6), allergic rhinitis (2/6), asthma (1/6), allergic contact dermatitis (2/6), CHE (1/6) Grover’s disease (1/6)	topical (6/6) and systemic (2/6) steroids, topical calcineurin inhibitor (4/6), topical Vitamin-D analogue (1/6), phototherapy (3/6), MTX (1/6), mycophenolic acid (2/6), ciclosporin (2/6)	ciclosporin (1/6)	Dupilumab 600 mg s.c., then 300 mg q2w	sustained complete response with BSA < 1% (5/6) at up to 2 years follow-up, temporary improvement (1/6)	[41]
Contact dermatitis	retrospective study (4)	15	55 (median, 28–72 range), females: 9/15	allergic contact dermatitis, hand involvement in 11/15	various type-IV sensitivities (15/15, including cocamidoylpropyl betaine in 40%, nickel in 33%), AD (11/15)	systemic steroid (15/15), ciclosporin (13/15), mycophenolic acid (8/15), MTX (2/15), apremilast (1/15), azathioprin (2/15), ustekinumab (2/15), etanercept (1/15)		Dupilumab 600 mg s.c., then 300 mg q2w	85% mean improvement in BSA (range 70–100%)	[43]
Contact dermatitis	case report (5)	1	M/55	allergic contact dermatitis	suspected type-IV sensitivity to chromate (no patch-testing performed), bronchitis, viral hepatitis C	topical steroids	Allergen exposure was continued.	Dupilumab 600 mg s.c. q2w	complete resolution of skin lesions at 8 wk	[44]
Contact dermatitis	case report (5)	1	M/61	allergic contact dermatitis	type-IV sensitivity to isobornyl acrylate (patch-testing performed), AD, asthma, diabetes mellitus	azathioprin (100 mg/d), topical steroid, antihistamines	Allergen exposure was continued.	Dupilumab 600 mg s.c., then 300 mg q2w	partial resolution of skin manifestations and pruritus at 7 wk, complete resolution at 16 wk	[45]
Contact dermatitis	case series (4)	2	F/65, M/51	allergic contact dermatitis	type-IV sensitivity to sesquiterpene lactones (patch-testing performed) (2/2), AD (1/2)	topical (2/2) and systemic (1/2) steroids, MMF (1/2), MTX (1/2), ciclosporin (1/2), azathioprin (150 mg/d, 2/2), ustekinumab (1/2)		Dupilumab 600 mg s.c., then 300 mg q2w	Partial improvement of skin lesions (2/2), dupilumab paused during winter without deterioration (2/2)	[46]
Contact dermatitis	case report (5)	1	M/54	allergic contact dermatitis, generalized eczema and hand/foot dermatitis	multiple type-IV sensitivities (including nickel, patch-testing performed), AD	topical steroid, phototherapy, acitretin		Dupilumab 600 mg s.c., then 300 mg q2w	Improvement of skin manifestations, repeated patch-testing remained positive	[47]
Contact dermatitis	case series (4)	3	F/20, F/52, F/53	allergic contact dermatitis, generalized (1/3), torso and extremities (2/3)	multiple type-IV sensitivities (3/3), type-I allergies (3/3), AD (1/3), allergic rhinitis (1/3)	systemic steroid (3/3), azathioprin (1/3), ciclosporin (1/3)		Dupilumab 600 mg s.c., then 300 mg q2w	≥90% improvement in BSA (3/3) after 6–12 wk	[48]
Contact dermatitis	case series (4)	6	47.5 (median, 26–67 range), females: 5/6	occupational irritant contact dermatitis (3/6), allergic contact dermatitis (3/6)	type-IV sensitivities (4/6), history of atopy (3/6)	topical (6/6) and systemic (1/6) steroids, phototherapy (3/6), alitretinoin (4/6), MTX (5/6)	Topical tacrolimus (1/6). Allergen/irritant exposure was continued (4/6).	Dupilumab 600 mg s.c., then 300 mg q2w	total clearance of skin lesions (5/6), partial remission (1/6)	[49]
Contact dermatitis	case report (5)	1	M/44	disseminated spongiotic dermatitis	type-IV sensitivities (nickel), nickel-containing stent, antiphospholipid syndrome, no AD or atopy	topical and systemic steroids, antihistamines, MMF (1.5 g/d)	systemic steroid, tapered	Dupilumab 600 mg s.c., then 300 mg q2w	significant improvement after 8 wk	[50]
Contact dermatitis	case series (4)	3	F/52, F/54, F/54	disseminated eczema including body and face	type-IV sensitivities (neomycin, fragrance and perfume (1/3); budesonide, limonene (1/3); shampoo (1/3)) AD (2/2)			Dupilumab 600 mg s.c., then 300 mg q2w	75%-improvement after 10 wk (1/3), full clearance after 8 wk (2/3)	[51]
Contact dermatitis	case report (5)	1	F/42	eczema on hands and arms	type-IV sensitivity (colophonium), AD	topical and systemic steroids, MTX, ciclosporin		Dupilumab 300 mg q2w	flare-up at former patch-testing sites (recall dermatitis), partial control at 10 wk	[52]
Contact dermatitis	case series (4)	2	F/83, F/69	disseminated eczema including body and extremitites	type-IV sensitivities (fragrance, propylene glycol (1/2); balsam of peru, propylene glycol (1/2))	topical and systemic steroids (2/2), MTX (15 mg/wk, 1/2), MMF (2 g/d, 1/2)	topical steroids and calcineurin inhibitors	Dupilumab 300 mg q2w	Significant improvement, BSA 2% after 4 mo (1/2) or 5% after 6 mo (1/2)	[53]
Contact dermatitis	retrospective study (4)	15	44 (mean, 18 SD), females: 12/17		AD (17/17)	4 modalities on average		Dupilumab	BSA improvement in 82%, 71%-reduction (mean, 42 SD), pruritus improved in 100%	[54]
Contact dermatitis	case report (4)	5	54 (median, 29–69 range), females: 2/5		AD or history of atopy (4/5), type-IV sensitivities (5/5, miscellaneous)	topical (5/5) and systemic (3/5) steroid, ciclosporin (4/5), mycophenolic acid (4/3), MTX (1/5), phototherapy (1/5)			80–100% improvement within 10–12 wk (5/5)	[55]
Contact dermatitis	retrospective study (4)	6	55.3 (mean, 4.9 SD), females: 4/6	generalized eczema	AD (6/6), type-IV sensitivities (multiple personal care products)		topical steroids, phototherapy (1/6)	Dupilumab 600 mg s.c., then 300 mg q2w	complete clearance of dermatitis (4/6), residual hand dermatitis (1/6), flares (1/6)	[56]
Contact dermatitis	case report (5)	1	F/43	pruritic rash on face and upper trunk after hair dyeing	type-IV sensitivity (p-phenylenediamine), dermatomyositis	topical steroids, antihistamines		Dupilumab 600 mg s.c., then 300 mg q2w	complete resolution after 6 wk	[57]
Contact dermatitis	case report (5)	1	M/52	facial eczema	no AD; type-IV sensitivities (sesquiterpene lactone, artichoke)	systemic steroids, ciclosporin		Dupilumab 600 mg s.c., then 300 mg q2w	partial response after 8 wk	[58]
Contact dermatitis	case report (5)	1	F/45	eczema of hands and arms	AD, allergic conjuncitivitis, type-IV sensitivity (rubber)	topical and systemic steroids, ciclosporin		Dupilumab 600 mg s.c. q2w	complete resolution of skin lesions at 18 wk, resolution of pruritus	[59]
Contact dermatitis	case report (5)	1	F/48	pruritic fissured hand eczema, eyelid eczema	asthma, no AD, type-IV sensitivities (p-phenylenediamine and others)	topical steroids		Dupilumab 600 mg s.c., then 300 mg q2w	no efficacy, discontinued	[60]
Contact dermatitis	case report (5)	1	M/54	vesiculobullous lesions of hands and feet	no AD; type-IV sensitivities (thiuram mix and others)	systemic steroids, ciclosporin		Dupilumab	no efficacy, discontinued after 12 mo	[61]
Dyshidrotic eczema	case series (5)	15	56 (mean, 32–76 range), females: 33%		no AD (15/15)	topical steroids (15/15), systemic immunosuppressive (7/15), phototherapy (3/15), psoriasis biologic (7/15)	ustekinumab (1/15, for concomitant psoriasis)	Dupilumab	Partial response (15/15) with reduced itch and skin lesions, complete clearance (6/15).	[63]
CHE	case report (5)	1	M/12	vesicular eczema of hands and feet with pruritus and painful lesions	AD	topical and systemic steroid, phototherapy, MTX (10 mg/m2/wk), ciclosporin (5 mg/kg biweekly)	topical mometasone and systemic prednisolone, tapered	Dupilumab 600 mg s.c., then 300 mg q2w	Gradual resolution of skin lesions at 4 wk, complete stable remission at 4 mo.	[64]
CHE	retrospective study (4)	19	55.9 (mean, 13.0 SD), females: 16/19	hyperkeratotic CHE (10/19), atopic CHE (4/19), allergic contact dermatitis (2/19), irritant contact dermatitis (1/19), pulpitis (1/19), vesicular hand eczema (1/19)	AD (6/19)	topical (19/19) and systemic (6/19) steroid, MTX (12/19), acitretin (6/19), alitretinoin (12/19), ciclosporin (5/19), azathioprin (11/19), MMF (7/19), psoriasis biologicals (7/19)		Dupilumab 600 mg s.c., then 300 mg q2w	Improvement of skin lesions and quality of life (14/19); no effect or deterioration (5/19). Effects were worse in hyperkeratotic CHE (*p* = 0.033 compared with all others)	[65]
CHE	prospective observational study (3)	72	45.2 (mean, 13.0 SD), females: 33.3%	chronic fissured HE (72.2%), recurrent vesicular HE (27.8%)	AD (100%), type-IV sensitivity (38.9%, patch testing performed), irritant contact dermatitis (22.2%), asthma (61.1%), allergic rhinitis (70.8%), allergic conjunctivitis (55.6%)	topical (100%) and systemic (81.9%) steroid, ciclosporin (93.1%), MTX (36.1%), azathioprin (26.4%), alitretinoin (13.9%), MMF (6.9%), systemic tacrolimus (2.8%)	Topical steroids or calcineurin inhibitors. No systemic immunosuppressive drugs (various washout intervals).	Dupilumab 600 mg s.c., then 300 mg q2w	HECSI −89% (95%-CI: −93.1 to −84.5) at wk 52, HECSI-90 met by 62.9%, no difference between HE-subtype or concomitant irritant contact dermatitis, improved quality of life	[66]
CHE	case series (4)	3	M/65, M/47, F/65	hyperceratotic HE (3/3)	no AD (3/3)	topical steroids (3/3), alitretinoin (3/3)	no topical treatment (2 wk washout)	Dupilumab 600 mg s.c., then 300 mg q2w	Improvement in itch and quality of life (3/3), complete clearance of skin lesions (2/3) at 16 wk, no effect on skin lesions (1/3)	[67]
CHE	case report (5)	1	M/43	occupational irritant hand dermatitis (compulsive hand washing, cleaning agents, steering wheel, handling coins)	no AD, no type-IV sensitvity (patch-testing performed)	topical and systemic steroids, phototherapy, acitretin (25 mg/d), MTX (15–20 mg/wk), antibiotics	topical steroid	Dupilumab 600 mg s.c., then 300 mg q2w	Improvement of HECSI (33 to 10 at 4 wk, 0 at 5 mo)	[68]
CHE	case report (5)	1	M/67	recurrent vesicular HE	no AD, no atopy, no type-IV sensitivity (patch-testing performed)	topical and systemic steroids, tar, phototherapy, MTX (25 mg/wk), azathioprine (150 mg/d), ciclosporin (200 mg/d)		Dupilumab 600 mg s.c., then 300 mg q2w	Improvement of skin lesions at 2 wk, complete clearance at 4 wk, sustained response for 3 mo follow-up	[69]
CHE	prospective observational study (3)	47	45.2 (mean, 20–69 range), females: 31.9%	chronic fissured HE (74.5%), recurrent vesicular HE (25.5%)	AD (100%), type-IV sensitivity (29.8%, patch testing performed)		Topical steroids or calcineurin inhibitors. No systemic immunosuppressive drugs (various washout intervals).	Dupilumab 600 mg s.c., then 300 mg q2w	Improvement of HECSI (in 45/47), HECSI-90 met by 32%, HECSI mean change −74.6% (95%-CI −67.9 to −81.2) at 16 wk. No difference between HE-types. No response in 2/45	[70]
CHE	retrospective study (4)	38	42.2 (mean, 18.4 SD), females: 23/38	various subtypes of HE including dyshidrotic eczema, atopic HE, contact dermatitis		systemic steroid (42%), MMF (32%), ciclosporin (16%)	Topical steroids	Dupilumab 600 mg s.c., then 300 mg q2w	Improvement in pruritus (96.7%, complete resolution in 26.7%), improvement in BSA (−15.9% mean) in ≥12 wk follow-up	[71]
CHE	case report (5)	1	F/50s	atopic HE	AD, asthma, rhinitis, multiple type-IV sensitivities (including nickel, cobalt)	topical and systemic steroids, phototherapy, alitretinoin (10–30 mg/d), ciclosporin (5 mg/kg/d), azathioprin (2.5 mg/kg/d), mycophenolic acid (1140 mg/d), tacrolimus (0.1 mg/kg/d), MTX (10–20 mg/wk)	prednisolone (7.5 mg/d)	Dupilumab 600 mg s.c., then 300 mg q2w	Improvement in HECSI from 244 to 11 (“almost clear”) in 16 wk	[72]
CHE	case series (4)	4	F/72, F/65, M/48	atopic HE (4/4)	AD (3/3), allergic rhinitis (2/3) multiple type-I sensitivities (1/3), no type-IV sensitvity (0/3, patch-testing performed)	topical (3/3) and systemic (2/3) steroids, topical (3/3) and systemic (1/3) calcineurin inhibitor, thalidomide (1/3), MTX (1/3), MMF (3/3), ciclosporin (2/3), apremilast (1/3), ustekinumab (1/3)		Dupilumab 600 mg s.c., then 300 mg q2w	Complete clearance (1/3), partial improvement (2/3) after 6–12 wk, sustained response at 3–8 mo follow-up	[73]
CHE	case series (4)	2	M/63, M38	dyshidrotic hand and foot eczema	asthma (1/2), no AD (0/2)	topical and systemic steroids (2/2), phototherapy (2/2), excimer laser (1/2), apremilast (2/2), MTX (2/2), efalizumab (1/2), etanercept (1/2), adalimumab (1/2), ixekizumab (1/2), ciclosporin (1/2), MMF (1/2)	ciclosporin (1/2)	Dupilumab 600 mg s.c., then 300 mg q2w	complete clearance after 8 wk (1/2) or 16 wk (1/2)	[74]
CHE	case report (5)	1	F/44	dyshidrotic hand and foot eczema	asthma, no type-IV sensibilities (patch-testing performed)	topical and systemic steroids, topical calcineurin inhibitors, antihistamines, excimer laser therapy,		Dupilumab 600 mg s.c., then 300 mg q2w	complete clearance after 8 wk	[75]
CHE	case report (5)	1	M/40s	dyshidrotic hand and foot eczema	no atopy	topical and systemic steroids, antifungals, ciclosporin		Dupilumab 600 mg s.c., then 300 mg q2w	Complete resolution after 3 wk	[76]
CHE	case series (4)	2	M/38 (2/2)	dyshidrotic hand (2/2) and foot (2/2) eczema	type IV-sensibilities (1/2; multiple)	topical and systemic steroids (2/2), apremilast (1/2), acitretin (1/2), phototherapy (1/2)	topical steroids (2/2), phototherapy (1/2)	Dupilumab 600 mg s.c., then 300 mg q2w	Near complete resolution after 1 (1/2) or 6 wk (1/2)	[77]
CHF	case report (5)	1	F/29	occupational irritant hand dermatitis	no atopy, no type-IV sensibilities (patch-testing performed)	topical seroids, topical calcineurin inhibitors, antihistamines		Dupilumab 600 mg s.c., then 300 mg q2w. Later q4w.	HECSI 116 to 15 after 4 wk	[78]
Eczematous eruption (αIL17R-induced)	case report (5)	1	M/62	generalized pruritic rash under brodalumab for psoriasis	psoriasis, AD, allergic rhinitis, latent tuberculosis infection	topical and systemic steroids, antihistamines	guselkumab; brodalumab discontinued	Dupilumab 600 mg s.c., then 300 mg q2w	Complete clearance of skin manifestations and itch at 8 wk	[79]
Eczematous eruption (αIL17 or -23 induced)	case series (4)	3	M/42, F/24, F/54	localized AD-like eczema	psoriasis (2/3), Crohn’s disease (1/3)	topical (3/3) and systemic (1/3) steroids, topical calcineurin inhibitors (3/3)	ixekizumab (1/3), ustekinumab (1/3), tildrakizumab (1/3) (all continued)	Dupilumab 600 mg s.c., then 300 mg q2w	Complete resolution (EASI 0) after 4 wk (1/3), 6 (1/3) or 7 mo (1/3)	[80]
Eczematous eruption of aging	case series (4)	15	75 (mean, SD: 8), females: 67%		no AD or atopy (15/15)	topical (15/15) and systemic steroids (11/15), topical calcineurin inhibitors (11/15), systemic immunosuppression (2/15), phototherapy (1/15)		Dupilumab 600 mg s.c., then 300 mg q2w	Improvement of skin lesions (15/15), BSA 20% (SD 15) to 2.6 (SD 4) in 2–8 wk. Sustained response in 7/15 after 8–12 mo follow-up	[81]
Eczematous eruption of aging	case report (5)	1	M/66	3-years history of generalized pruritic rash excluding head and neck	no atopy; type-IV allergy to ciprofloxacin	topical and systemic steroids, MTX (17.5 mg/wk), MMF (1 g/d), phototherapy		Dupilumab 600 mg s.c., then 300 mg q2w	Complete clearance of skin manifestations and itch at 8 wk, sustained at 4 mo follow-up	[82]
Eczematous eruption (CVID)	case report (5)	1	F/59	Recurrent generalized pruritic rash	CVID, type-IV sensitivity (chrome, nickel, colophonium, mercapto-mix, thiomersal)	topical and systemic steroids, ciclosporin (150 mg/d), IVIg		Dupilumab 600 mg s.c., then 300 mg q2w	Control of itch in 4 wk. Complete resolution of skin manifestations (EASI 41.10 to 1.20) in 8 wk.	[83]
Autoeczema-tization in chronic stasis dermatitis	case report (5)	1	M/80	generalized pruritic papules and eczematous patches, venous stasis dermatitis of lower extremity	no type-IV sensitivity (patch-testing performed)	topical and systemic steroids, compression therapy	compression therapy	Dupilumab 600 mg s.c., then 300 mg q2w	Improvement of itch (-7 NRS) and reduced lower extremity edema at 10 wk. New psoriasiform dermatitis, dupilumab discontinued.	[84]

Reports of eczema other than AD treated with dupilumab were identified via database search. Evidence levels 1 through 5 were assigned to each report according to the Oxford Centre for Evidence Based Medicine and denoted in parentheses after the study type. AD, atopic dermatitis; BSA, body surface area; CHE, chronic hand eczema; CVID, common variable immunodeficiency; DLQI, dermatology life quality index; EASI, eczema area and severity index; HE, hand eczema; HECSI, hand eczema severity index; MMF, mycophenolate mofetil; mo, month; MTX, methotrexate; NRS, numerical rating scale; q2w, biweekly; s.c., subcutaneous; SD, standard deviation; wk, week.

Chronic hand eczema, also known as dermatitis (CHE), can be a clinical form of AD (atopic HE), ACD or ICD, but unrelated etiologies are found as well. Frequent clinical phenotypes include chronic hyperkeratotic/fissured and recurrent dyshidrotic/vesicular HE among others. Reports of a total of 162 patients demonstrated clinical effectiveness of dupilumab in various subtypes of CHE for patients that had failed various topical and systemic therapies including most frequently topical and systemic steroids, topical calcineurin inhibitors, retinoids, ciclosporin A and phototherapy (Table 1) [63,64,65,66,67,68,69,70,71,72,73,74,75,76,77,78]. A prospective study following 72 patients with chronic fissured or vesicular HE for 52 weeks showed a mean reduction of 89% of the hand eczema severity index (HECSI, 95%; confidence interval 93.1–84.5%) and a 90% improvement of HECSI met by 62.9% with no difference between clinical subtypes [66]. Another retrospective study of 19 patients indicated significantly smaller effects of dupilumab in patients with hyperkeratotic CHE compared to all other investigated subtypes (*p* = 0.033) [65]. Likewise, a lack of response to dupilumab or even deterioration was reported in a total of 6 cases, all of which had hyperkeratotic CHE [65,67]. Two phase 2 placebo-controlled RCTs evaluating effects of dupilumab were found with one being currently active with patients with severe CHE (DUPSHE, NCT04512339) and one currently recruiting patients with moderate to severe CHE (DUPECZEMAIN, NCT03861455).

Localized or generalized eczema can develop in response to drugs in the form of drug-induced eczematous eruption (EE) typically described for TNFα-inhibitors. Dupilumab treatment of EE induced by anti-IL-17 or -23 agents (tildrakizumab, brodalumab, ixekizumab and ustekinumab) given for underlying psoriasis or Crohn’s disease led to complete clearance within months in all 4 reported patients [79,80]. EE of aging is an exclusion diagnosis and was treated with dupilumab leading to sustained improvement of skin lesions and itch in a total of 16 patients without a history of AD or atopic diathesis (Table 1) [81,82]. Furthermore, one record of a patient with EE associated with a common variable immunodeficiency (CVID) demonstrated complete resolution of skin manifestations within 8 weeks [83]. Secondary generalized eczematization in a patient with underlying chronic venous stasis dermatitis was treated with dupilumab which led to significant improvement of itch; however, new psoriasiform lesions developed subsequently and dupilumab was discontinued [84].

### 3.2. Chronic Pruritus and Prurigo

Chronic pruritus imposes a high disease burden and can be refractory to multiple treatment regimens including topical and systemic steroids as well as systemic antipruritic medications such as antihistamines, antidepressants, antiemetics, opioid-antagonists, cannabinoids and anticonvulsants. Etiologies are diverse and include AD and atopy as well as other dermatologic, systemic/metabolic or mental diseases. Th2-mediated inflammation with complex interactions between neurons, keratinocytes and immune cells via chemokines, neuropeptides, alarmins and proteases are described. This includes direct effects on sensory neurons by IL-4 and -13 [85]. Itch-scratch cycles can sustain pruritus and induce skin lesions (Figure 1B). Most notably, dupilumab was approved for prurigo nodularis (PN) in adults. Additionally, two case reports showed efficient use of dupilumab in weight-adjusted dosing schemes in pediatric patients with PN (Table 2) [86,87].

Chronic pruritus of unknown origin (CPUO) or chronic idiopathic pruritus refers to itch lasting more than 6 weeks without an underlying medical condition and can be accompanied by skin lesions. Opposed to skin lesions found in prurigo, CPUO manifestations are secondary. Several reports demonstrated efficient itch control by dupilumab measured by numerical scales, including a case series of 15 cases with a mean itch numerical rating scale (NRS)-reduction of 7 points (SD 1.9) as well as a series of 4 cases with a mean reduction of 8.75 points (SD 1.26). Notably, 26 out of 27 reported patients had no AD. In two cases, dupilumab was withdrawn after only 4 weeks and a sustained response was reported for a 20 weeks follow-up period (Table 2) [8,88,89,90]. Currently, the placebo-controlled RCT LIBERTY-CPUO-CHIC (NCT05263206) is ongoing and recruiting.

Dupilumab further demonstrated efficiency in pruritus with various defined etiologies. This included 6 cases with uremic pruritus [8,91] and one case with cholestatic pruritus (Table 2) [92]. A clinical exploratory study evaluating dupilumab in cholestatic pruritus is currently recruiting (NCT04256759). Efficient treatment of localized pruritus was reported for a case of neuropathic brachioradial pruritus [93] and a case of anal and genital pruritus (Table 2) [94].

Reactive perforating collagenosis (RPC) is characterized by umbilicated nodules with a central keratotic plug and is frequently associated with chronic kidney disease. Reports of six cases showed partial improvement of itch NRS as well as resolution of skin lesions under dupilumab treatment. Notably, three patients had no history of AD or atopy (Table 2) [95,96,97,98,99].

### 3.3. Bullous and Acantholytic Dermatoses

Dermatoses with sub- or intraepidermal cleft formation and blistering can be caused by autoantibody deposition and subsequent recruitment of effector cells [100], or by inherited defects of structural proteins of the dermoepidermal junction (DEJ) or the epidermis [101].

The most frequent acquired bullous dermatosis is bullous pemphigoid (BP) caused by IgG autoantibodies against BP180 and/or BP230 [102,103]. Autoantibody binding at the DEJ leads to complement fixation and, most prominently, the activation of neutrophils and eosinophils inducing itch and the formation of plaques and tense blisters (Figure 1C). IL-4 and -13 as well as other Th2-cytokines were found in higher concentrations in sera of patients with BP, as well as in blister fluid [103]. Peripheral eosinophilia is often seen in blood and in inflamed skin. Further, total IgE concentrations in BP sera are frequently increased and correlate with disease severity [104]. Thus, therapeutic efficacy of dupilumab was hypothesized due to inhibition of eosinophil chemotaxis and activation directed by Th2-associated chemokines as well as reduction of IgG and IgE synthesis by B cells stimulated by Th2-responses.

Several case reports showed successful treatment of BP-patients that were resistant to previous standard of care treatments or had contraindications for high-dose systemic steroids or immunosuppressants leading to complete clinical remissions defined by resolution of skin lesions and pruritus (Table 3) [105,106,107,108,109,110,111,112,113,114,115,116]. This included one pediatric patient [117]. Efficient dupilumab mono-therapy inducing clinical remission was reported in 3 cases [118,119,120]. However, some reports showed only partial or no response [114]. In addition, 4 cases of checkpoint inhibitor-induced BP were treated efficiently with dupilumab, a favorable medication as strong immunosuppression needs to be avoided due to the underlying malignancy [121,122,123,124]. Two patients with BP presumably triggered by COVID-19 vaccinations were also treated efficiently with dupilumab [120,125]. Most noticeably, two retrospective cohort studies compared co-therapy of dupilumab with conventional therapy against conventional therapy alone (comprised of systemic high-dose steroids and immunosuppressants) and found shorter median time to disease control, more rapid decline of itch measured by NRS and disease activity (measured by the BP disease activity index, BPDAI), higher quality of life (measured by the dermatology life quality index, DLQI) as well as lower cumulative doses of steroids and immunosuppressants in a total of 28 patients [126,127]. A large retrospective study (NCT05649579) and a placebo-controlled RCT evaluating dupilumab in BP (LIBERTY-BP, NCT04206553) are currently recruiting. 

More rare subtypes of BP treated efficiently with dupilumab include pemphigoid gestationis [128], lichen planus pemphigoides [129,130], Brunsting–Perry cicatricial pemphigoid [131,132] and pemphigoid nodularis [133]. Additionally, a case of linear IgA-dermatosis was efficiently treated with dupilumab [134].

By contrast, pemphigus vulgaris (PV), the most frequent intraepidermal autoimmune blistering disease [135], is caused by autoantibodies against desmoglein-3 (Dsg-3), and eosinophilia is seen far less frequently than in BP. Two reports, however, showed efficiency of dupilumab as mono-therapy or as an add-on therapy to strong immunosuppression in a recalcitrant PV case (Table 3) [136,137]. This could be attributed to the finding of Dsg-3 reactive Th2-cells in PV-patients that might stimulate Dsg-3 autoantibody production by B lymphocytes [138]. However, data regarding dupilumab in pemphigus diseases is limited.

**Table 3:** Bullous and acantholytic dermatoses.

Disease	Study Type	n	Sex/Age	Medical History	Prior Systemic Therapies	Concomitant Systemic Medication	Therapy	Response	Reference
BP	case series (4)	7	74 (median, 63–88 range)	hypertension (4/7), diabetes (2/7), MDS (1/7)	none (5/7), steroid + ciclosporin (1/7), tofacitinib + omalizumab (1/7)	none (1/7), methylprednisolone 0.5–0.6 mg/kg/d with reduction (5/7), prednisolone 0.5 mg/kg/d with reduction (1/7)	Dupilumab 600 mg s.c. initially, then 300 mg q2w for 16 wk	Total BPDAI reduced to 2 (median, IQR 6 to 0), *p* < 0.0001 at 16 wk, Reduction of BP180 and BP230 ab, also IgE. 4 stopped dupilumab, no relapse. 2 prolonged dosing with 300 mg q3-4w, no relapse. 1 relapse after taper of dupilumab, controlled again with 300 mg q2w. 1 relapse after glucocorticoid taper, controlled with dupilumab.	[105]
BP	case series (4)	2	F/53, M/78		prednisolone 0.5–0.8 mg/kg/d	prednisolone 20 mg/d	Dupilumab 600 mg s.c. initially, then 300 mg q2w. Withdrawal after 2 months	Sustained clinical remission	[106]
BP	case series (4)	2	M/72, M/88		methylprednisolone (2/2), MTX (1/2)		Dupilumab 600 mg s.c. initially, then 300 mg q2w	Relief of pruritus, improvement of lesions at 2 wk follow-up	[107]
BP	case series (4)	3	2F, 1M	psychiatric disorders (1/3), Hepatitis B (2/3), gastric ulcers (1/3)	steroids (max. equivalent to 2.5 mg/kg/d prednisone, 2/3), IVIG (2/3), cyclophosphamide (1/3), MTX (1/3), ciclosporin (1/3), none (1/3)	prednisone (0.75 mg/kg/d) + cyclophosphamide (1/3), methylprednisolone + MTX + ciclosporin (1/3), none (1/3)	Dupilumab 600 mg s.c. initially, then 300 mg q2w	Relief of pruritus (3/3), improvement of skin lesions (2/3), clinical remission (1/3)	[108]
Vesicular BP	case report (5)	1	M/32	pulmonary tuberculosis	high-dose systemic steroids	prednisolone 30 mg/d, isoniazide, rifampicin, ethambutol	Dupilumab 600 mg s.c. initially, then 300 mg q2w	Clinical remission, no relapse of tuberculosis	[109]
BP (non-bullous)	case report (5)	1	M/74	diabetes mellitus, hypertension	high-dose systemic steroids		Dupilumab 600 mg s.c. initially, then 300 mg q2w	Sustained clinical remission from 4 wk	[110]
BP	case report (5)	1	F/61		methylprednisolone (max. 0.5 mg/kg/d), azathioprine (100 mg/d)	methylprednisolone, azathioprine (100 mg/d)	Dupilumab 600 mg s.c. initially, then 300 mg q2w	Resolution of pruritus and cessation of blister development within 1 month. Sustained clinical remission.	[111]
BP	case report (5)	1	M/80		prednisone 40 mg/d, doxycycline 200 mg/d, mycophenolate mofetil 1.000 mg/d, niacinamide 1.500 mg/d	prednisone, doxycycline	Dupilumab 600 mg s.c. initially, then 300 mg q2w	Sustained clinical remission	[112]
BP	case report (5)	1	M/70	obesity, diabetes mellitus, hypertension	dapsone (150 mg/d), MTX (7.5 mg/wk s.c.), mycophenolate mofetil (2 g/d), omalizumab (300 mg s.c. q4w)	mycophenolate mofetil, omalizumab	Dupilumab 600 mg s.c. initially, then 300 mg q2w	Reduced itch NRS (0/10) and cessation of new lesions after 3 months, sustained clinical remission	[113]
BP	case series (4)	13	78 (median, 53–91 range)		none (1/13), steroids (9/13), MMF (2/13), rituximab (2/13), IVIG (3/13), azathioprine (1/13), nicotinamide (3/13), doxycycline (4/13), MTX (4/13)	none (7/13), MTX (3/13), prednisone with taper (3/13)	Dupilumab 600 mg s.c. initially, then 300 mg q2w or qw	Sustained clinical remission (7/13), relief of pruritus (12/13), no response (1/13)	[114]
BP	case report (5)	1	M/89	diabetes mellitus	doxycycline (200 mg/d), nicotinamide (1000 mg/d), MMF (2 g/d), prednisone (10 mg/d), omalizumab	prednisone (2.5 mg/d), MMF, doxycycline, nicotinamide	Dupilumab 600 mg s.c. initially, then 300 mg q2w	Relief of pruritus at 2 wk, resolution of BP lesions at 7 wk. Sustained clinical remission at 1 year.	[115]
BP	case report (5)	1	M/86	PN, type 2 diabetes mellitus	methylprednisolone, azathioprine, doxycycline	methylprednisolone, doxycycline; both discontinued after 4 mo	Dupilumab 600 mg s.c. initially, then 300 mg q2w	Complete remission after 4 mo, sustained under dupilumab-monotherapy for 10 mo follow-up	[116]
BP	case report (5)	1	F/17		doxycycline, methylprednisolone 75 mg/d, prednisolone, rituximab (q2w, later q4w), plasmapheresis (13 sessions total), IVIG (2g/kg q4w)	steroids, rituximab, IVIG	Dupilumab 600 mg s.c. initially, then 300 mg q2w	Complete blister resolution, undetectable BP180 (initially 574 U/mL)	[117]
BP	case report (5)	1	M/85	suspected AD, asthma, ulcerative colitis	tofacitinib and omalizumab for suspected AD, ineffective	none	Dupilumab 600 mg s.c. initially, then 300 mg q2w	Sustained clinical remission at 6 mo	[118]
BP	case report (5)	1	M/80		prednisone	none	Dupilumab 600 mg s.c. initially, then 300 mg q2w	Sustained clinical remission at 10 mo	[119]
BP, COVID-vaccination induced	case report (5)	1	M/78	diabetes mellitus, hypertension, hyperlipidemia	prednisone, doxycycline	none	Dupilumab 600 mg s.c. initially, then 300 mg q2w	Sustained clinical remission	[120]
BP (ICI-induced)	case report (5)	1	M/76	Melanoma St. IV (adjuvant Nivolumab 480 mg i.v. q4w for 6 mo)	methylprednisolone (0.6 mg/kg/d), doxycycline (200 mg/d), nivolumab discontinuation	methylprednisolone, tapered	Dupilumab 300 mg s.c. q2w	Sustained clinical remission	[121]
BP (ICI-induced)	case report (5)	1	F/59	Cervical cancer St. IIB (adjuvant Pembrolizumab for 5 wk prior)	methylprednisolone (1 mg/kg/d), doxycycline (200 mg/d), niacinamide (1 g/d), dapsone (75 mg/d), pembrolizumab discontinuation	methylprednisolone (0.75 mg/kg/d), tapered	Dupilumab 600 mg s.c. initially, then 300 mg q2w	Cessation of new blister formation at 2 mo, severe flare after discontinuation, sustained clinical remission after re-initiation of dupilumab	[122]
BP (ICI-induced)	case report (5)	1	F/79	Melanoma St. II (adjuvant Nivolumab for 11 mo prior)	prednisone, doxycycline, dapsone		Dupilumab 600 mg s.c. initially, then 300 mg q2w	Sustained clinical remission reached at 4 wk	[123]
BP and GD (ICI-induced)	case report (5)	1	M/73	Metastatic renal cell carinoma (ipilimumab/nivolumab treatment), autoimmune thyreoiditis	steroids, doxycycline, dapsone		Dupilumab 600 mg s.c. initially, then 300 mg q2w	Complete resolution of skin lesions and pruritus of both BP and GD	[124]
BP, COVID-vaccination induced	case report (5)	1	F/91	hypertension, chronic kidney failure	prednisone, azathioprine, rituximab (1 cycle)	prednisone with tapering	Dupilumab 600 mg s.c. initially, then 300 mg q2w	Resolution of pruritus and BP lesions after 2 months	[125]
BP	Retrospective cohort study (3)	20 vs. 20	72 (median, 54–86 range)	hypertension (30%), cardiovascular disease (15%), diabetes mellitus (20%), chronic renal insufficiency (15%), neurologic disorder (25%), interstitial lung disease (25%), tumor (5%)	no prior therapy (17/20 cases, 20/20 controls)	methylprednisolone (<0.4 mg/kg/d)	Dupilumab 600 mg s.c. initially, then 300 mg q2w vs. 0.4 mg/kg/d methylprednisolone	Shorter median time to disease control (14 vs. 19 days, *p* = 0.043), lower cumulative dose of steroid (*p* < 0.01)	[126]
BP	Retro-spective cohort study (3)	8 vs. 16	64.5 (median, 22–90 range)	cardiovascular disease (3/8), neurologic disorder (1/8), hyperlipidemia (3/8), tumor (2/8)		methylprednisolone (0.6 mg/kg/d) + azathioprine (2 mg/kg/d) with reduction	Dupilumab 600 mg s.c. initially, then 300 mg q2w vs. methylprednisolone (0.6 mg/kg/d) + azathioprine (2 mg/kg/d) with reduction	More rapid decline of itch NRS (*p* = 0.034) and BPDAI (*p* = 0.0308), shorter median time to cessation of new blisters (8 vs. 12 days, *p* = 0.028), lower cumulative dose of methylprednisolone (*p* = 0.036), lower cumulative dose of azathioprine (*p* = 0.0048)	[127]
Pemphigoid gestationis	case report (5)	1	F/37	20 wk of gestation, g5, p4	prednisone (0.5 mg/kg/d)	prednisone, tapered	Dupilumab 600 mg s.c. initially, then 300 mg q2w	Decline of BP18 autoantibodies, clearance of skin lesions, sustained clinical remission postpartum. Newborn without skin lesions	[128]
LPP	case report (5)	1	M/69	Lichen planus mucosae (20 years)	prednicolone (50 mg/d)	prednisolone, tapered	Dupilumab 600 mg s.c. initially, then 300 mg q2w	Sustained clinical remission reached at 2 wk, normal BP180 ab	[129]
LPP	case report (5)	1	M/18	AD	dexamethasone (8 mg/d), MMF (1 g/d)	none	Dupilumab 600 mg s.c. initially, then 300 mg q2w	Partial remission at 4 wk, clinical remission reached at 15 wk and sustained after discontinuation of dupilumab	[130]
Brunsting-Perry Pemphigoid	case report (5)	1	M/71		prednisone, MMF	none	Dupilumab 600 mg s.c. initially, then 300 mg q2w	Partial clearance of bullae and erosions after 6 wk, persistent occasional bullae, mild pruritus, scarring	[131]
Brunsting-Perry Pemphigoid	case report (5)	1	F/63	AD, allergic rhinitis	rituximab, steroids, MMF, MTX, dapsone, doxycycline, nicotinamide		Dupilumab 300 mg s.c. q2w	Partial remission	[132]
Pemphigoid nodularis	case report (5)	1	F/76	hypertension, diabetes, obesity, and atrial fibrillation	none	none	Dupilumab 600 mg s.c. initially, then 300 mg q2w	Sustained clinical remission reached at 4 mo	[133]
LAD	case report (5)	1	M/63	AD, allergic rhino-conjunctivitis	methylprednisolone, azathioprine, dapsone (100 mg/d), colchicine (3 mg/d)		Dupilumab 600 mg s.c. initially, then 300 mg q2w	Sustained clinical remission reached at 4 wk	[134]
Pemphigus vulgaris	case report (5)	1	F/41		steroids	none	Dupilumab 600 mg s.c. initially, then 300 mg q2w	Clearance of oral lesions at 6 wk, sustained clinical remission	[136]
Pemphigus vulgaris	case report (5)	1	M/35		steroids, IVIG	steroids, IVIG	Dupilumab 600 mg s.c. initially, then 300 mg q2w	Partial remission with PDAI -55 at 6 wk	[137]
DEB-Pr (*COL7A1* mutation)	case report (5)	1	F/52	no AD	antihistamines, promethazine, cannabis, St. John’s wort	none	Dupilumab 600 mg s.c. initially, then 300 mg q2w	Improvement of itch NAS and quality of life (measured by DLQI), improvement of skin lesions at 12 wk	[139]
DEB-Pr (*COL7A1* mutation)	case series (4)	2	M/15, F/27	asthma (1/2), ADHD (1/2)	steroids (1/2), antihistamines (2/2), ciclosporine (2/2), MMF (1/2), thalidomide (2/2), lenalidomide (1/2), omalizumab (1/2), tofacitinib (1/2), gabapentin (1/2), pregabalin (1/2), naltrexone (1/2), melatonine (1/2), clonidine (1/2), ondansetron (1/2), antidepressants (2/2), phototherapy (2/2), dermabrasio (1/2)	none	Dupilumab 600 mg s.c. initially, then 300 mg q2w (1/2) or qw (1/2)	Sustained improvement of itch (-3.5 and -7 NRS), improvement of skin findings, improvement of sleep	[140]
DEB-Pr (*COL7A1* mutation)	case report (5)	1	M/39		dapsone, cyproheptadine	none	Dupilumab 600 mg s.c. initially, then 300 mg q2w	Improvement of itch at 2 wk, cessation of new lesions at 4 wk, partial remission at 9 mo	[141]
DEB-Pr (*COL7A1* mutation)	case report (5)	1	F/10		steroids	none	Dupilumab 600 mg s.c. initially, then 300 mg q2w	Improvement of itch NAS and quality of life (measured by DLQI)	[142]
DEB-Pr (*COL7A1* mutation)	case report (5)	1	F/43	frequent bacterial skin infections		none	Dupilumab 600 mg s.c. initially, then 300 mg q2w	Improvement of itch, no more episodes of skin infections	[143]
Hailey-Hailey	case report (5)	1	F/22		ciclosporine (5 mg/kg/d)	none	Dupilumab 300 mg s.c. q2w	Resolution of skin lesions after 4 mo	[144]
Hailey-Hailey	case series (4)	3	F/56, M/52, F/59		antihistamines (3/3), acitretin (3/3), steroids (3/3), MTX (2/3), ciclosporine (2/3), hydroxychloroquine (2/3), naltrexone (2/3), apremilast (3/3), fluconazole (2/3), tetracyclines (3/3), dapsone (3/3), oxybutynin (2/3), MMF (1/3), local laser ablation (1/3)	none	Dupilumab 600 mg s.c. initially, then 300 mg q2w	Improvement in affected body surface area (BSA) and quality of life (2/3), no improvement (1/3)	[145]
Hailey-Hailey	case series (4)	3	F/50s, M/50s, M/70s		antihistamines (3/3), isotretinoin (1/3), etanercept (1/3), steroids (1/3), acitretin (2/3), naltrexone (2/3), antibiotics (1/3), ciclosporine (1/3), local botulinum toxin (1/3)	none	Dupilumab 600 mg s.c. initially, then 300 mg q2w	Reduction of size and thickness of skin lesions	[146]
GD	case report (5)	1	M/71	Metastatic renal cell carinoma (ipilimumab/nivolumab treatment)	pulsed prednisone (60 mg/d max.), antihistamines, gabapentin, aprepitant, topical phototherapy		Dupilumab 600 mg s.c. initially, then 300 mg q2w	Complete resolution of skin lesions and pruritus at 3 mo; sustained after restart of ipilimumab/nivolumab	[147]
GD	case series (4)	3	M/70s, F/50s, M/70s		steroids (3/3), azathioprine (1/3), MTX (2/3), gabapentin (1/3), topical phototherapy (1/3), acitretin (1/3)		Dupilumab 600 mg s.c. initially, then 300 mg q2w	Complete resolution of skin lesions and pruritus within 2 mo (3/3)	[148]
GD	case report (5)	1	M/77	CRSwNP	steroids	pulsed steroid	Dupilumab 600 mg s.c. initially, then 300 mg q2w	Complete resolution of skin lesions and pruritus at 14 wk	[149]

Reports of hereditary or autoimmune bullous and acantholytic dermatoses treated with dupilumab were collated after a database search. Evidence levels 1 through 5 were assigned to each report according to the Oxford Centre for Evidence Based Medicine and denoted in parentheses after the study type. ab, antibody; AD, atopic dermatitis; ADHD, attention deficit hyperactivity disorder; BP, bullous pemphigoid; BPDAI, bullous pemphigoid disease activity index; BSA, body surface area; CRSwNP, chronic sinusitis with nasal polyps; DEB-Pr, dystrophic epidermolysis bullosa, pruritic subtype/epidermolysis bullosa pruriginosa; GD, Grover’s disease; ICI, immune-checkpoint inhibitor; IQR, interquartile range; IVIG, intravenous immunoglobulins; LAD, linear IgA-dermatosis; LPP, lichen planus pemphigoides; MDS, myelodysplastic syndrome; MMF, mycophenolate mofetil; mo, month; MTX, methotrexate; NRS, numerical rating scale; PDAI, pemphigus disease activity index; PN, prurigo nodularis; q2w, biweekly; q3w, every 3 weeks; q4w, every 4 weeks; qw, weekly; s.c., subcutaneous; wk, weeks.

Dystrophic epidermolysis bullosa (DEB) is a hereditary subepidermal blistering disease caused by mutations in the gene for collagen 7 (*COL7A1*). DEB can be associated with intense pruritus highly refractory to anti-inflammatory (steroids, phototherapy) and antipruritic treatments and is termed epidermolysis bullosa pruriginosa (DEB-Pr). Dupilumab showed efficient reduction of itch measured by NAS and despite the hereditary origin of the disease also led to improvement of the skin findings in 6 patients with DEB-Pr that were resistant to extensive previous therapy regimes (Table 3) [139,140,141,142,143]. This reflects a putative Th2-mediated component of the disease and the role of itch–scratch cycles possibly similar to PN.

The most frequent hereditary intraepidermal blistering disease is Hailey–Hailey disease (HHD) often caused by mutations in *ATP2C1* that lead to disrupted development of desmosomes of the epidermis and subsequent acantholysis. A total of seven patients were treated with dupilumab after extensive ineffective previous therapies including immunosuppressants, retinoids, antipruritic medications or dermabrasion with significant improvement of skin lesions, itch and quality of life in six patients (Table 3) [144,145,146]. One patient did not respond to dupilumab. HHD-caused skin barrier defects might give rise to secondary Th2-mediated local inflammation possibly inhibited by treatment with dupilumab.

Grover’s disease (GD) is characterized histologically by acantholysis; yet, no disease mechanism is known. A total of five patients were reported with complete resolution of skin lesions and pruritus by dupilumab, including one patient with concomitant BP [124,147,148,149].

### 3.4. Alopecia Areata

Despite being one of the most common forms of nonscarring hair loss, treatment options for alopecia areata (AA, Figure 1D) are still limited. Topical immunosuppression or immunomodulation (including topical steroids, diphenylcyclopropenone, squaric acid, photodynamic therapy), minoxidil as well as systemic immunosuppression, are used with varying effects. Recently, the JAK1/2-inhibitor baricitinib was approved for severe AA in adults. Disease mechanisms in AA are not fully elucidated and possibly heterogeneous: multiple studies suggested a major role of interferon (IFN)-γ-mediated Th1-responses, while others highlighted Th2-mediated effects and regulatory T cell (Treg) deficiency [150]. Notably, patients with AD have a high susceptibility to develop AA, with one study finding a 26-fold increased chance compared to healthy individuals [151], underlining the role of Th2-skewed responses in AA.

Several case reports and case series showed clinical efficiency of dupilumab in 51 patients with AA, including patients with alopecia totalis (AT) and universalis (AU). Most of the patients treated had AD and reported multiple failed previous therapies. One case of incontinentia pigmenti-associated scalp alopecia was reported. Clinical responses were mostly measured by the severity of alopecia tool (SALT) with a score < 10 defined as complete response (Table 4) [152,153,154,155,156,157,158,159,160,161,162,163,164,165,166,167,168,169,170,171]. Noticeably, 13 pediatric patients were treated effectively with dupilumab [172,173,174,175,176,177]. However, 15 of a total of 66 reported patients did not experience clinical improvement; notably, one case series with 10 patients found no significant improvements throughout the investigated cohort [178].

Additionally, a phase 2 randomized clinical trial (NCT03359356) [179] compared weekly injections of 300 mg dupilumab versus placebo and showed improvement in SALT at week 24 (*p* = 0.049) and week 48 (*p* < 0.0001) as well as 30% improvement in SALT (SALT30) at week 48 reached by 32.5% versus 20% (*p* = 0.067) and a 50% improvement in SALT (SALT50) at week 48 reached by 22.5% versus 15% (*p* = 0.02). Interestingly, high serum levels of IgE (>200 U/mL) as well as a medical history of AD or family history of atopy predicted a better outcome.

Conversely, several reports showed novel development of AA after commencing therapy with dupilumab for AD (exemplarily, [180]). Possible predictors of clinical outcomes (IgE serum levels, disease severity, concomitant atopy, age at onset, sex) have to be considered. Currently, a multicenter RCT is registered and not yet recruiting (NCT05551793).

### 3.5. Chronic Urticaria

Urticaria that lasts for more than 6 weeks is defined as chronic urticaria and can have a broad spectrum of etiologies and provocation factors causing recurrent urticae and swellings (Figure 1E). Dupilumab may exert an inhibitory effect on mast cells by hindering IL-4 driven proliferation and chemotaxis, IL-4 regulated expression of FcεRI and by lowering IgE-production from B cells; also, IL-4 blockade can desensitize vascular structures towards histamine and decrease mast cell-mediated anaphylaxis [181].

Chronic spontaneous urticaria (CSU) entails the absence of identified causes of urticaria and can be highly debilitating and recalcitrant to standard treatments. A total of 14 patients were reported that had failed standard therapies with antihistamines, omalizumab and/or ciclosporin and were subsequently treated with dupilumab (12/14) or a combination of dupilumab and omalizumab (2/14) (Table 5) [182,183,184,185,186,187,188,189]. Complete sustained resolutions were reported in 10/14 patients by clinical evaluation and the 7-day urticaria activity score (UAS7) or urticaria control test (UCT); a UAS7 of 0 was reported in 5 of these cases. In total, 3/14 patients had a partial response; one patient ended therapy due to financial reasons. Notably, 9/14 patients had AD or other signs of atopy. The placebo-controlled phase 3 clinical trial LIBERTY-CSU CUPID Study A investigated 138 patients with antihistamine-refractory CSU and found an 8.5 points-higher reduction of UAS7 in the dupilumab-treated cohort (*p* = 0.0003) as well as significantly reduced itch and hives at week 24; strikingly, the effects of dupilumab were independent of baseline serum IgE (NCT04180488) [190,191]. The phase 3 LIBERTY-CSU CUPID Study B enrolled 83 omalizumab-refractory CSU patients and was closed after interim analysis due to the lack of significant differences (NCT04180488). The phase 2 clinical trial DUPICSU (NCT03749135) has not published results, yet.

Besides CSU, dupilumab treatment in cases with chronic inducible urticaria with identified triggers were reported. A patient with cold urticaria experienced complete remission and tolerance to cold water under therapy with dupilumab [192]; one patient with exercise-induced cholinergic urticaria had a complete remission after 8 weeks with dupilumab [193]; further, a patient with adrenergic urticaria was efficiently treated with a combination of propranolol and dupilumab [194]. Three placebo-controlled RCTs are registered for evaluation of dupilumab in chronic inducible urticaria: A phase 2 RCT is recruiting adults with cholinergic urticaria (CHED, NCT03749148) and a phase 3 RCT for adults with cold urticaria is currently active (LIBERTY-CINDU CUrIADS, NCT04681729). Furthermore, a phase 3 RCT is currently recruiting children (2–12 years) with cold urticaria or CSU (LIBERTY-CSU/CINDU CUPIDKids, NCT05526521).

### 3.6. Netherton Syndrome and Other Hereditary Skin Diseases

Mutations in the gene *SPINK5* in patients with Netherton syndrome causes dysfunction of the skin-expressed serine protease LEKTI that can lead to severe pruritus, skin inflammation and increased IgE. Subsequent epidermal remodeling makes patients susceptible to atopic manifestations resembling AD (Figure 1F) as well as skin infections. Other typical findings are ichthyosis (type linearis circumflexa) and hair abnormalities (trichorrhexis invaginata). Dupilumab treatment for patients with Netherton syndrome was reported in a total of 16 cases (Table 6) [195,196,197,198,199,200,201,202,203,204,205]. All cases showed prompt resolution of itch measured by NRS and an improvement of skin lesions evaluated by EASI or BSA within 3 months. In patients with hair abnormalities, three cases reported improved hair growth while two reported no effect. The response was sustained in 14/16 cases for various follow-up periods, while two patients experienced a relapse after 8 or 20 weeks that was not sufficiently controlled by a dose increase and led to withdrawal of dupilumab [195,202]. Currently, a placebo-controlled RCT is recruiting (NS-DUPI, NCT04244006).Other congenital ichthyoses can lead to skin findings similar to AD by impaired skin barrier functions. Clinical improvement of ichthyosis in patients with concomitant AD treated with dupilumab was reported in a case of lamellar ichthyosis [206] and erythrodermic ichthyosis [207]. A partial response to dupilumab with improved skin findings but persistent itch was reported in one case of peeling skin syndrome 1 [208]. A case of ichthyosiform erythroderma caused by trichothiodystrophy with *ERCC2*-mutation experienced complete remission of skin lesions and pruritus with dupilumab [209]. An exploratory study in children with congenital ichthyoses compares effects of dupilumab and other biologicals to symptomatic treatment and is currently recruiting (NCT04996485).

One report of a patient with neurofibromatosis type 1 treated with dupilumab for concomitant AD showed a size reduction of preexisting neurofibromas and stable size and number of neurofibromas for a follow-up of 1.5 years [210].

*TTC7A* deficiency is a rare congenital disease that leads to intestinal atresia and various immune defects; one patient with pruritic eczema and ichthyosis as well as immunologic findings suggesting vancomycin-induced linear IgA-dermatosis was treated efficiently with dupilumab and experienced complete sustained resolution of itch and skin findings [211].

Mutations in *FOXP3* lead to dysfunction of Tregs and severe clinical findings termed IPEX syndrome often associated with various inflammatory skin diseases. One case of a patient with pruritic eczema due to IPEX syndrome was treated efficiently with dupilumab with sustained resolution of skin findings after a range of ineffective aggressive immunosuppressive therapies as well as bone marrow transplantation [212].

A patient with X-linked agammaglobulinemia that resulted in frequent skin infections and AD-like eczema experienced complete remission with dupilumab [213].

### 3.7. Eosinophilic Dermatoses

Peripheral and tissue eosinophilia is known to be promoted by IL-4, -5 and -13 by stimulating eosinophil trafficking and inducing other eosinophil chemoattractants [214]. Several dermatoses are specifically characterized by eosinophilic skin infiltrates or result from systemic eosinophilia.

Hypereosinophilic syndrome (HES) is a difficult to treat hematologic disease characterized by idiopathic blood eosinophilia that can present with pruritic eczema and other organ manifestations. A total of 11 patients with HES, 7 of which had skin manifestations, were treated with dupilumab after failure of systemic steroids. Of note, 2/7 patients reported a history of atopy. Improvement of skin lesions to various degrees was reported in 5/7 patients, one non-atopic individual experienced complete remission (Table 7) [215,216,217].

Hematologic malignancies can be associated with pruritic rashes with skin eosinophilia, a condition termed eosinophilic dermatosis of hematological malignancy (EDHM). We found records of four patients with EDHM treated with dupilumab after failure of systemic steroids, three of which had underlying chronic lymphatic leukemia and one small lymphocytic lymphoma. In two of the reported patients, strikingly, no eosinophilia was found in the blood. All four patients reported rapid complete clearance of skin manifestations within 4–6 weeks (Table 7) [8,218,219,220].

Hyper-IgE syndromes can be caused by a variety of mutations and patients typically present with generalized eczema and peripheral eosinophilia. Clinically and mechanistically, eczema in hyper-IgE syndromes have a large overlap with AD. We found records of a total of 12 patients with hyper-IgE syndrome presenting with generalized pruritic eczema that was treated with dupilumab. Of those, seven patients had a mutation of *STAT3*, three of *DOCK8* and one of *ZNF341*. A complete remission of pruritus and skin findings were reported in 10/12 patients andtwo patients showed partial remissions (Table 7) [221,222,223,224,225,226,227,228,229,230,231].

Further reports were found for Kimura’s disease with 4 patients treated with dupilumab and 3/4 experiencing complete and one partial remission [232,233,234,235]. Four cases of steroid-refractory papuloerythroderma Ofuji treated with dupilumab all reported complete resolution of skin lesions and pruritus [236,237,238]. Two cases of highly steroid- and immunosuppressant-refractory Well’s syndrome reported complete resolutions [239,240], as did two cases of the related erythema annulare eosinophilicum [241,242]. One case of eosinophilic fasciitis reported resolution of skin induration clinically and in MRI-studies under dupilumab treatment [243]. Further, one case of angiolymphoid hyperplasia with eosinophilia refractory to IL-5 inhibitors treated with dupilumab reported sustained resolution of pruritic skin lesions even after discontinuation of the therapy [244].

### 3.8. Connective Tissue Disorders

Several systemic or localized connective tissue inflammatory diseases are accompanied by pruritus. Th2-associated inflammation has been found in some diseases, but Th1-pathways are more prominent in systemic collagenoses such as lupus erythematosus.

Dupilumab showed reduction of skin lesions and pruritus in one case of extragenital lichen sclerosus (Table 8) [245]; further, a phase 2 placebo-controlled study is recruiting patients for evaluating dupilumab in localized scleroderma (DupiMorph, NCT04200755).

No effects have, however, been shown for systemic collagenoses, e.g., in a case of dermatomyositis with severe pruritus, dupilumab was ineffective [246]. Notably, some reports showed newly developed lupus erythematosus in AD patients treated with dupilumab. In contrast, a case of steroid-refractory IgG4-related disease and concomitant AD with retroperitoneal fibrosis and pruritic skin manifestations showed resolution of fibrosis and skin lesions on dupilumab [247].

One report showed increased expression of Th2-associated transcripts (IL-4 receptor, IL-13) in keloid tissue in an individual with severe AD. Dupilumab was evaluated in a total of 12 reported cases with keloids (Table 8). One case showed size reduction of the keloid [248], one case with a highly pruritic keloid reported reduction of itch [249]; however, the remaining 10 cases did not report any effects of dupilumab or deterioration [250,251]. Two clinical trials evaluating dupilumab in keloids are currently recruiting (NCT04988022, NCT05128383).

### 3.9. Other Inflammatory Skin Diseases

Chronic photodermatoses including chronic actinic dermatosis (CAD) and actinic prurigo are induced by exposure to UV-radiation and can resemble photosensitive AD. Reports showed efficacy of dupilumab in 16 cases of CAD (Table 8) [252,253,254,255,256,257,258] as well as in a pediatric patient with actinic prurigo [259], leading to higher minimal erythema doses in repeated UV-phototesting in some cases.

Graft-versus-host disease (GVHD) after hematopoietic stem cell transplantation can show clinical and histologic features of AD; a case series of 4 pediatric patients showed complete resolution of GVHD under treatment with dupilumab, while one patient did not respond [260].

One case found complete resolution of a severe palmoplantar pustulosis with dupilumab after lacking disease control with secukinumab in a non-atopic individual [261]. Psoriasiform dermatoses, however, have been implicated as adverse effects of dupilumab in other reports.

Lichen planus was treated efficiently with dupilumab in three cases, leading to resolution of skin manifestations and pruritus (Table 8) [8,262,263]. Conversely, also lichenoid dermatoses were reported as adverse effects of dupilumab.

Three cases of patients with hidradenitis suppurativa (HS) and concomitant AD showed remission of the previously uncontrolled HS after having failed systemic antibiotics and in one case adalimumab (Table 8) [264,265,266]. High AD-disease activity might impose an additional trigger to flares of HS, however, HS itself is mainly associated with Th1/Th17-responses.

One case of generalized granuloma annulare refractory to adalimumab showed a sustained partial response [267], however, emergence of granulomatous drug reactions was also reported under dupilumab therapy.

Lichen amyloidosus is characterized by extracellular deposits of amyloid proteins in the dermis with often intense pruritus. Reports of 4 patients with lichen amyloidosus and concomitant AD treated with dupilumab showed flattening of dermal papules and reduction of itch (Table 8) [268,269], a partial resolution was also observed in one non-atopic individual after failure of therapy with benralizumab [270].

Food allergies can be associated with AD. One case report of a patient that had suffered an anaphylactic shock after ingestion of corn and had detection of specific IgE against corn extract showed no reaction in a later oral provocation after 12 weeks of dupilumab therapy for AD [271]. A phase 2 clinical trial evaluating dupilumab in 24 patients with peanut allergy showed a tolerance to oral challenge after 24 weeks of therapy in 8.3% (NCT03793608). Another phase 2 study in patients with peanut allergy is completed and has not yet reported (NCT03682770); one was terminated due to COVID-19 (NCT04462055). Currently, a phase 2 study for patients with milk allergy is recruiting (NCT04148352).

### 3.10. Cutaneous Lymphoma

Cutaneous T cell lymphoma (CTCL), including mycosis fungoides and Sézary syndrome, is frequently accompanied by highly refractory pruritus that drastically affects quality of life, especially in palliative settings (Figure 1G). Dupilumab showed reduction of itch as a supportive care treatment in 10 patients (Table 9) [272,273,274,275,276]. However, 7 of those experienced disease progression with two reported deaths; an average length of initial improvement of 1.9 months was reported for 6 patients. Other reports of 8 patients showed no response, primary worsening or new development of CTCL under dupilumab [274,275,276,277,278,279,280,281,282]. Possible mechanisms could be increased binding of IL-13 to IL-13RA2 expressed on lymphoma cells that is not inhibited by dupilumab. Thus, dupilumab could merely be considered a supportive care strategy for symptom control.

One patient with cutaneous B cell pseudolymphoma showed complete remission with dupilumab [283].

Further, one patient with a treatment-refractory generalized lichenoid drug eruption associated with mogamulizumab given for treatment of underlying MF reported complete resolution of the drug rash with dupilumab [284].

## 4. Discussion

This systematic review summarizes the existing evidence for treatment with dupilumab in other dermatologic conditions than atopic dermatitis and prurigo nodularis.

A high level of evidence (level 2 according to the OCEBM) was found for alopecia areata with a phase 2 RCT reporting significant improvement of the disease score SALT at week 24 and 48; however, conflicting case reports showed paradoxical reactions or lack of effects (15/66 reported patients).

Further, a high level of evidence (level 2) was found for antihistamine-refractory chronic spontaneous urticaria with a phase 3 RCT showing a significantly higher reduction of the disease score UAS7 compared with placebo in 138 treated patients. Anecdotal evidence was found for other forms of chronic urticaria.

For bullous pemphigoid, a medium level of evidence (level 3) was found based on multiple cohort studies. Dupilumab could become more relevant in checkpoint inhibitor-induced BP for patients with underlying malignancy.

A medium level of evidence (level 3) was also found for chronic hand eczema with a large prospective study showing an average 89% reduction of the disease score HECSI at 52 weeks. Numerous reports also showed efficacy of dupilumab in allergic contact dermatitis; however, the culprit allergen seemed to affect responses.

Lower levels of evidence (level 4) were found for nummular eczema, chronic pruritus of unknown origin and Netherton syndrome. Anecdotal evidence (level 5) based on individual reports was shown for eczematous eruptions, cholestatic or uremic pruritus, perforating collagenosis, other pemphigoid diseases than BP and pemphigus diseases, epidermolysis bullosa dystrophica, Hailey–Hailey disease, Grover’s disease, congenital ichthyoses and other genodermatoses, eosinophilic dermatoses such as Kimura’s disease, chronic photodermatoses, graft-versus-host disease and food allergies.

For symptomatic treatment of cutaneous lymphoma, the reports were ambivalent and some showed progression or novel development of the underlying disease.

Strengths of this systemic review are the multifaceted comprehensive evaluation of dupilumab effects in a range of heterogeneous dermatologic conditions in order to give a broad overview. However, the evidence found was scarce or conflicting for many diseases and mainly based on individual reports; statistical meta-analysis was thus not feasible and larger cohort sizes are needed. In addition, many of the reported conditions can be considered multicausal with unidentified factors confounding the responses to dupilumab. In addition, dupilumab was frequently given as second- or third-line treatment in refractory patients.

As a drug with favorable safety profile as well as established and efficient clinical use for a broad range of patient populations [285], dupilumab holds significant promise for amending treatment options for a plethora of dermatologic conditions. With numerous clinical trials ongoing, we expect future expansion of the use of dupilumab in dermatology.

## Figures and Tables

**Table 2:** Chronic pruritus and prurigo.

Disease	Study Type	n	Sex/Age	Medical History	Prior Therapies	Concomitant Therapy	Therapy	Response	Reference
PN	case report (5)	1	F/9	no AD, normal IgE	topical steroids, antihistamines, phototherapy, ciclosporin (4 mg/kg/d), MTX (7.5–10 mg/wk), psychiatric interventions		Dupilumab 200 mg s.c., then 100 mg q2w	Decreased pruritus at 4 wk, resolution of skin lesions at 3 mo	[86]
PN	case report (5)	1	M/16	no AD, no family history of atopy	antihistamines, topical steroids, ciclosporin (5 mg/kg/d)	ciclosporin, tapered	Dupilumab 600 mg s.c., then 300 mg q2w	Resolution of skin lesions at 3 mo, reduction of itch NRS, improved quality of life (by DLQI)	[87]
CPUO	case series (4)	6	60.5 (median, 43–78 range), female: 2/6	asthma (1/6), hypereosinophilia (2/6), type-IV sensitivity (3/6), cardiovascular disease (4/6), nephrotic syndrome (1/6), tumor (1/6), depression (1/6), diabetes mellitus (1/6), sarcoidosis (1/6)	antihistamines (5/6), topical (6/6) and systemic (6/6) steroids, MMF (4/6), topical calcineurin inhibitor (3/6), phototherapy (1/6), mirtazapine (1/6), gabapentin (1/6), aprepitant (1/6), azathioprin (1/6)		Dupilumab 600 mg s.c., then 300 mg q2w (5/6) or q4w (1/6)	Improvement in pruritus and skin lesions (6/6)	[88]
CPUO	case series (4)	15	68.7 (mean, SD 12.6), female: 67%	Exclusion of patients with AD or atopy, other primary dermatologic disorder or systemic disease-causing itch		topical steroids	Dupilumab 600 mg s.c., then 300 mg q2w	Improvement of itch NRS mean -7.0 (SD 1.9) at various follow-up intervals (mean 19 mo, interquartile range 10–26)	[89]
CPUO	case series (4)	2	F/68, M/86	no AD or atopy (2/2), hypereosinophilia (1/2), cardiovascular disease (1/2)	topical steroids (2/2), antihistamines (2/2), phototherapy (1/2), MMF (1/2), gabapentin (1/2), pregabalin (1/2)		Dupilumab 600 mg s.c., then 300 mg q2w	Resolution of pruritus at 4 or 5 mo (2/2) measured by worst itch-NRS	[90]
CPUO	case series (4)	4	65 (median, 56–66 range), female: 3/4	AD (1/4)	topical steroids, topical calcineurin inhibitors		Dupilumab 600 mg s.c., then 300 mg q2w	Reduction of itch NRS -8.75 (mean, SD 1.26) at wk 12; withdrawal of dupilumab at 4 wk with sustained response at wk 20 (2/4)	[8]
Uremic pruritus	case series (4)	5	64 (median, 57–78 range), female: 2/5	no AD or atopy (5/5)	topical steroids, topical calcineurin inhibitors	hemodialysis (1/5)	Dupilumab 600 mg s.c., then 300 mg q2w	Reduction of itch NRS -6.4 (mean, SD 3.51) at 12 wk	[8]
Uremic pruritus	case report (5)	1	F/61	Allergic rhinitis, hypertension, polycystic kidney disease, renal transplant (aged 53)	phototherapy, doxepin, aprepitant, pregabalin, naltrexone, mirtazapin, topical steroid, topical anesthetic	phototherapy, gabapentin (tapered)	Dupilumab 600 mg s.c., then 300 mg q2w	Reduction of itch NRS to 0 at 6 mo, improvement in quality of life (DLQI 1 at 6 mo)	[91]
Cholestatic pruritus	case report (5)	1	M/44	Primary sclerosing cholangitis and autoimmune hepatitis	topical steroids, topical retinoids, bile acid sequestrants, anticonvulsants, antihistamines, doxepin, phototherapy	phototherapy	Dupilumab 600 mg s.c., then 300 mg q2w	Improvement in pruritus (-8 NRS), resolution of skin lesions, improvement in quality of life (DLQI)	[92]
Brachioradial pruritus	case report (5)	1	F/53	fibromyalgia, cervical disc protrusion, scoliosis	topical steroid, topical doxepin, antihistamines, gabapentin, pregabalin	topical steroid, topical anesthetic, amitriptyline (discontinued after 4 wk)	Dupilumab 600 mg s.c., then 300 mg q2w	95% improvement of pruritus at 12 wk, sustained response for 8 mo	[93]
Anal and genital pruritus	case report (5)	1	M/62	ADHD, depression, asthma, lumbal spine degeneration, multiple food allergies and type-IV sensitivity	topical antiinfectives, topical and systemic steroids, topical calcineurin inhibitors, topical doxepin, topical capsaicin, antihistamines, gabapentin, MMF (3 g/d)		Dupilumab 600 mg s.c., then 300 mg q2w	95% resolution of itch and resolution of skin lesions at 4 wk, sustained response at 12 mo	[94]
RPC	case report (5)	1	M/66	no AD or atopy; chronic kidney disease, hypertension, diabetes mellitus	phototherapy, nalfurafine		Dupilumab 600 mg s.c., then 300 mg q2w	Reduction of itch NRS from 10 to 2 at 4 wk, resolution of skin lesions	[95]
RPC	case report (5)	1	M/59	diabetes mellitus, pulmonary tuberculosis	systemic steroids, hydroxychloroquine		Dupilumab 600 mg s.c., then 300 mg q2w	Improvement of itch and skin lesions after 2 wk	[96]
RPC	case report (5)	1	F/20	Wilson’s disease, liver transplant (aged 15)	systemic steroids (20 mg/d), systemic tacrolimus (5 mg/d) due to transplant	Immune-suppressive therapy after transplant was continued	Dupilumab 600 mg s.c., then 300 mg q2w	Clearance of pruritus within 2 wk, clearance of active skin lesions at 10 wk	[97]
RPC	case report (5)	1	F/40s	AD	topical and systemic steroids, phototherapy, antihistamines, ciclosporin	phototherapy	Dupilumab 600 mg s.c., then 300 mg q2w	Improvement in pruritus after 2 mo, resolution of skin lesions afer 12 mo	[98]
RPC	case series (4)	2	M/71, M/70	AD (2/2), cardiovascular disease (2/2), diabetes mellitus (2/2)	antihistamines (2/2), topical steroids (2/2), phototherapy (1/2)	none (2/2)	Dupilumab 600 mg s.c., then 300 mg q2w	Partial reduction of pruritus and reduction of skin lesions at 6 or 12 wk	[99]

Reports of dupilumab used to treat chronic pruritus and defined other types of prurigo than PN in adults were obtained by database search. Evidence levels 1 through 5 were assigned to each report according to the Oxford Centre for Evidence Based Medicine and denoted in parentheses after the study type. AD, atopic dermatitis; ADHD, attention deficit hyperactivity disorder; CPUO, chronic pruritus of unknown origin; DLQI, dermatology life quality index; MMF, mycophenolate mofetil; mo, month; MTX, methotrexate; NRS, numerical rating scale; PN, prurigo nodularis; q2w, biweekly; q4w, every 4 weeks; RPC, reactive perforating collagenosis; s.c., subcutaneous; SD, standard deviation; wk, week.

**Table 4:** Alopecia areata.

Disease	Study Type	n	Sex/Age	Medical History	Prior Systemic Therapies	Prior Topical Therapies	Concomitant Therapy	Therapy	Response	Reference
AU	case report (5)	1	F/21	AD	UVB phototherapy, tofacitinib			Dupilumab 300 mg q2w	Regrowth of hair on scalp, eyebrowes and eyelashes at 2 mo. Only one alopecic patch left at 4 mo	[152]
AU	case report (5)	1	F/49	AD, allergic rhinitis, asthma, polysensitization	betamethasone (0.5 mg/d), ciclosporin			Dupilumab 600 mg s.c., then 300 mg q2w	Partial regrowth of terminal hairs and body hairs, regrowth of eyebrows, sustained for 1, 5 years	[153]
AA (AU)	case report (5)	1	F/34	AD, asthma	steroids			Dupilumab 600 mg s.c., then 300 mg q2w	Complete regrowth within 10 mo	[154]
AA (AT)	case report (5)	1	M/30	AD	steroids, MTX, ciclosporine	steroids, phototherapy		Dupilumab 600 mg s.c., then 300 mg q2w	Complete regrowth within 3 mo	[155]
AA (AT)	case report (5)	1	F/68	no AD, no family history of AD	steroids, ciclosporine	photodynamic therapy, steroids, squaric acid		Dupilumab 600 mg s.c., then 300 mg q2w	Improvement in SALT at wk 2 (-92), complete regrowth within 3 mo	[156]
AA	case report (5)	1	F/33	AD, trichotillomania				Dupilumab 600 mg s.c., then 300 mg q2w	Partial regrowth within 3 wk, complete regrowth within 19 wk	[157]
AA (AT 3/7, AU 1/7)	case series (4)	7	40.0 (mean, 33–52 range), females: 2/7	AD	steroids (5/7)	steroids (5/7), diphenylcyclopropenon (4/7), squaric acid (1/7), phototherapy (2/7)		Dupilumab 600 mg s.c., then 300 mg q2w	Complete regrowth (SALT < 10, 2/7), partial response (4/7), no response (1/7)	[158]
AA	case series (4)	2	M/38, M/32	AD (2/2), allergic rhinoconjuncitivis (2/2), asthma (2/2)	ciclosporine (2/2), azathioprine (1/2)	phototherapy (2/2)		Dupilumab 600 mg s.c., then 300 mg q2w	Complete regrowth at wk 21 or 22 (2/2)	[159]
AA	case report (5)	1	M/49	AD	steroids, azathioprine, ciclosporine, MTX	phototherapy		Dupilumab 600 mg s.c., then 300 mg q2w	Complete regrowth at 3 mo	[160]
AA	case report (5)	1	M/44	AD	steroids	steroids	steroids	Dupilumab 600 mg s.c., then 300 mg q2w	Complete regrowth at 3 mo (SALT -53)	[161]
AA (AU)	case report (5)	1	F/35	AD, chronic urticaria	steroids, omalizumab, ciclosporin	steroids		Dupilumab 600 mg s.c., then 300 mg q2w	Complete regrowth at 12 mo	[162]
AA (AU)	case report (5)	1	M/28	AD, asthma	ciclosporin (5 mg/kg/d), MTX (20 mg/wk)			Dupilumab 600 mg s.c., then 300 mg q2w	Complete regrowth (SALT -80.4)	[163]
AA	case report (5)	1	M/21	AD	ciclosporin (5 mg/kg/d)			Dupilumab 600 mg s.c., then 300 mg q2w	Complete regrowth (SALT 8.2) at 16 wk. Report also includes another case of AA developed as an adverse effect of dupilumab	[164]
AA (AU)	case report (5)	1	F/49	AD	pulsed prednisone, MTX (20 mg/wk)	steroids, calcineurin inhibitors, phototherapy		Dupilumab 600 mg s.c., then 300 mg q2w	Complete regrowth at 8 mo	[165]
AA (AT)	case report (5)	1	F/28	AD		steroids, phototherapy		Dupilumab 600 mg s.c., then 300 mg q2w	Nearly complete regrowth at 2–3 mo	[166]
AA (AU)	case report (5)	1	F/44	AD	pulsed methylprednisolone (500 mg/d max.)	steroids, phototherapy	topical steroid	Dupilumab 600 mg s.c., then 300 mg q2w	Complete regrowth at 10 m. Patient developed psoriasis as uncommon adverse effect of dupilumab.	[167]
AA (AT)	case report (5)	1	M/47	AD	ciclosporin, MTX			Dupilumab 600 mg s.c., then 300 mg q2w	partial regrowth at 8 wk	[168]
AA (AU)	case report (5)	1	M/65	AD		steroids and calcineurin inhibitors		Dupilumab 600 mg s.c., then 300 mg q2w	partial regrowth at 10 mo	[169]
AA (3/4)	case series (4)	4	F/40, F/51, F/54, F/42	AD (4/4), asthma (2/4), allergic rhinitis (1/4), Basedow’s disease (1/4)	steroids			Dupilumab 600 mg s.c., then 300 mg q2w	Complete regrowth within 4 mo (3/4), novel development of AA (1/4) after 8 mo	[170]
AA	case report (5)	1	F/33	no AD	steroids	steroids, calcineurin inhibitors, minoxidil, squaric acid, calcipotriene, platelet-rich plasma, tofacitinib cream		Dupilumab 600 mg s.c., then 300 mg q2w	Near complete regrowth of hairs within 6 mo, SALT reduction (81.3 to 2.4). Relapse at 6 mo after discontinuation of dupilumab	[171]
Inconti-nentia pigmenti with curvi-linear scalp alopecia	case report (5)	1	F/6	Incontinentia pigmenti, AD	antihistamines			Dupilumab	Regrowth of terminal hairs at 10 wk	[172]
AT	case report (5)	1	M/16	AD, asthma, Type-I allergy to eggs	montelukast			Dupilumab 600 mg s.c., then 300 mg q2w	Complete regrowth of hairs within 8 mo, sustained for 3 years	[173]
AA	case series (4)	6	M/12, M/7, F/7, F/8, F/7, F/12	AD (6/6), asthma (2/6), food allergies (3/6)	steroids (2/6), minoxidil (1/6)	steroids (6/6), minoxidil (2/6), anthralin (1/6), tofacitinib (1/6)	none (2/6), topical (1/6) or systemic (3/6) minoxidil, topical tofacitinib (2/6), topical (1/6) or systemic (1/6) steroids	Dupilumab	Complete regrowth (4/6) with reduction of SALT to 0, partial regrowth (1/6) with 73% SALT reduction after 2 years, no response (1/6)	[174]
AA (7/14 AU)	case series (4)	16	13.5 (median, 8–19 range)	AD (14/14),	steroids (12/14), MTX (9/14), tofacitinib (3/14)	steroids (14/14), anthralin (5/14), retinoid (7/14), tofacitinib (4/14), squaric acid (2/14), minoxidil (3/14)	none (8/14), topical (2/14) or systemic (2/14) tofacitinib, oral MTX (1/14), topical steroids (3/14), topical minoxidil (2/14), spironolactone (1/14)	Dupilumab 300 mg s.c. q2w	Initial worsening (4/14, SALT average worsening of 11.3), complete regrowth (3/14), partial response (1/4; SALT average reduction of 33.3 after 12 mo), no response (2/14), 8/14 had no active disease at baseline	[175]
AA (AT)	case report (5)	1	F/4	AD	antihistamines	steroids, calcineurin inhibitors		Dupilumab 200 mg s.c. q2w	Complete regrowth within 4 mo	[176]
AA (AT)	case report (5)	1	F/13	AD	pulsed prednisone (50 mg/d max.), MTX (15 mg/wk max.)	squaric acid, anthralin		Dupilumab 600 mg s.c., then 300 mg q2w	Partial regrowth at 11 mo	[177]
AA (AT 5/10, AU 3/10)	case series (4)	10	43.71 (mean, SD 8.22), females: 4/10	AD (10/10)	ciclosporin (7/10)	steroids (10/10), diphenylcyclo-propenone or squaric acid (7/10),		Dupilumab 600 mg s.c., then 300 mg q2w	Non-significant improvement of SALT (SALT30 32.5%, with elevated IgE 53.8%) at wk 48, no complete responses	[178]
AA (AT/AU in 32.5%)	RCT (2)	40 vs. 20	41.6 (mean, SD 13.8), females: 75%	AD (42.5%, active AD 12.5%), family history of atopy (45%). Other severe, progressive or uncontrolled disease was excluded			none; use of systemic immunosuppression, topical steroids or topical calcineurin inhibitors was excluded	Dupilumab 300 mg qw vs. Placebo	Improvement in SALT at wk 24 (*p* = 0.049) and wk 48 (*p* < 0.0001), 30%-improvement in SALT at wk 48 in 32.5% vs. 20% (*p* = 0.067), 50%-improvement in SALT at wk 48 in 22.5% vs. 15% (*p* = 0.02), high IgE levels (>200 U/mL) or AD/family history of atopy predicted better outcome	[179]

Reports of patients with alopecia areata treated with dupilumab were collected from databases and ClinicalTrials.gov. Evidence levels 1 through 5 were assigned to each report according to the Oxford Centre for Evidence Based Medicine and denoted in parentheses after the study type. AA, alopecia areata; AD, atopic dermatitis; AT, alopecia totalis; AU, alopecia universalis; mo, month; MTX, methotrexate; q2w, biweekly; qw, weekly; RCT, randomized controlled trial; s.c., subcutaneous; SALT, severity of alopecia tool; wk, weeks.

**Table 5:** Chronic urticaria.

Disease	Study Type	n	Sex/Age	Presentation	Medical History	Prior Therapies	Concomitant Medication	Therapy	Response	Reference
Adrenergic urticaria	case report (5)	1	M/18	Papules surrounded by vasoconstricted halos, triggered by heat, stress and exercise	POTS	antihistamines, montelukast, dapsone, omalizumab	propranolol (120 mg/d)	Dupilumab q4w	Complete resolution	[169]
CSU	case report (5)	1	F/68	recurrent wheals and episodes of facial angioedema, onset 6 mo prior	chronic renal insufficiency	antihistamines, omalizumab, systemic steroids	systemic steroids (discontinued), antihistamines (tapered), omalizumab (450 mg q4w, later reduced to 300 mg q4w)	Dupilumab 600 mg s.c., then 300 mg q2w	Complete disease control after 6 wk (no new wheals or angioedema, UCT 16)	[182]
CSU	case report (5)	1	M/44	pruritic wheals and papules, angioedema		antihistamines, ciclosporin, phototherapy, systemic steroids, omalizumab		Dupilumab 600 mg s.c., then 300 mg q2w	Complete disease control after first application (UAS7 0, no more pruritus, wheals or angioedema), stable for 2 years	[183]
CSU	case report (5)	1	F/47	4-month history of hives	AD, multiple type-I allergies	antihistamines, topical and systemic steroid, omalizumab (450 mg q4w)	omalizumab 450 mg q4w, fexofenadine 720 mg/d, prednisolone 12.5 mg/d (tapered)	Dupilumab 600 mg s.c., then 300 mg q2w	Partial control of CSU with UAS7 21 improved to 7, remission of AD with EASI 2.6, improved quality of life (DLQI 3) at 12 mo	[184]
CSU	case series (4)	2	F/63, F/52	wheals and lip angioedema for 3 (1/2) and 11 (1/2) years	AD (1/2)	antihistamines (2/2), omalizumab (2/2), ciclosporin (2/2), montelukast (1/2), MTX (1/2)	none (1/2), antihistamines (1/2)	Dupilumab 600 mg s.c., then 300 mg q2w	Complete remission (2/2) after 8 wk, sustained at latest follow-up (5 or 23 mo)	[185]
CSU	case series (4)	6	35.5 (median, 18–50 range), females: 3/6		AD (6/6), asthma (2/6), autoimmune thyroiditis (1/6),	antihistamines, omalizumab	antihistamines (6/6), topical steroid or calcineurin inhibitor (5/6), montelukast (1/6), gabapentin (1/6), dapsone (1/6)	Dupilumab 600 mg s.c., then 300 mg q2w	Follow-up for to 34 mo. Complete remission with UAS7 = 0 (4/6). Uncontrolled disease under dupilumab, omalizumab added (1/6), dupilumab discontinued for financial reasons and relapse (1/6)	[186,187]
Cold urticaria	case report (5)	1	M/28	cold urticaria since childhood (positive ice-cube skin test)	severe AD, multiple type-I sensitizations, type-IV sensitization to nickel	antihistamines, systemic steroid, omalizumab, ciclosporin		Dupilumab 600 mg s.c., then 300 mg q2w	85% reduction of EASI, 86% reduction of itch NRS, DLQI 0, ice-cube test negative, no wheals after cold-water exposure	[192]
Cholinergic urticaria	case report (5)	1	M/26	pruritic wheals after physical exercise	no atopy	antihistamines, systemic steroid, montelukast, omalizumab q4w	antihistamines, discontinued after 8 wk	Dupilumab 600 mg s.c., then 300 mg q2w	Complete remission after 8 wk	[193]
CSU	case series (4)	2	6–17 years		atopy (1/2)	antihistamines, omalizumab, ciclosporin		Dupilumab 300 mg s.c. q2w	UAS7 = 0 after 4 wk (1/2), significant improvement of symptoms after 12 wk (1/2)	[188]
CSU	case report	1	M/31	3-year history of recurrent itchy wheals		antihistamines, omalizumab		Dupilumab 600 mg s.c., then 300 mg q2w	UAS7 = 0 at wk 16, sustained for 42 wk follow-up	[189]

Records of patients with different entities of chronic spontaneous or inducible urticaria treated with dupilumab were acquired from the databases. Evidence levels 1 through 5 were assigned to each report according to the Oxford Centre for Evidence Based Medicine and denoted in parentheses after the study type. AD, atopic dermatitis; CSU, chronic spontaneous urticaria; DLQI, dermatology life quality index; EASI, eczema area and severity index; mo, month; MTX, methotrexate; NRS, numerical rating scale; POTS, postural orthostatic tachycardia syndrome; q2w, biweekly; q4w, every 4 weeks; s.c., subcutaneous; UAS7, urticaria activity score summed over 7 days; UCT, urticaria control test; wk, weeks.

**Table 6:** Netherton syndrome and other hereditary skin diseases.

Disease	Study Type	n	Sex/Age	Medical History	Prior Therapies	Concomitant Therapy	Therapy	Response	Reference
Lamellar ichthyosis	case report (5)	1	M/22	AD, asthma, Stargardt’s syndrome	Acitretin (10 mg/d), MTX (10 mg/wk)		Dupilumab 300 mg s.c. q2w	Clinical improvement of ichthyosis and AD after 3 mo	[188]
Netherton syndrome	case series (4)	4	3.25 (median, 2–4.5 range), females: 2/4	ichthyosis linearis circumflexa (4/4), trichorrhexis invaginata (4/4) growth delay (4/4), food allergies (1/4)		topical steroid or topical caldineurin inhibitor	Dupilumab 400 mg s.c., then 200 mg q2w	Temporary response with improvement in pruritus and skin lesions at 6 wk. Deterioration after 8 wk, itch and skin findings worse than at baseline by wk 10.	[195]
Netherton syndrome	case report (5)	1	F/29	asthma, ichthyosis, hair abnormalities, polycystic ovary syndrome	topical and systemic steroids, antihistamines, phototherapy, antibiotics	none	Dupilumab 300 mg s.c. q2w	Reduced itch (NRS -8), resolution of skin lesions (EASI –16), higher quality of life (DLQI –15) in 12 wk. Hair anomalities remained.	[196]
Netherton syndrome	case report (5)	1	F/42	Ichthyosis linearis circumflexa, trichorrhexis invaginata	topical steroids, antihistamines		Dupilumab 600 mg s.c., then 300 mg q2w	Reduced itch (NRS -10), resolution of skin lesions (EASI –21), higher quality of life (DLQI –24) in 8 wk. Improvement of ichthyosis. Hair regrowth.	[197]
Netherton syndrome	case report (5)	1	F/41	Ichthyosis linearis circumflexa with erythroderma	topical and systemic steroids, antibiotics, ciclosporin	topical steroid	Dupilumab 600 mg s.c., then 300 mg q2w, later qw	Reduced erythema and scaling, relapse after 5 mo and insufficient control at dose-increase to 300 mg qw, withdrawn after 6 mo	[198]
Netherton syndrome	case report (5)	1	M/26	ichthyosis and erythroderma, sleeping disorder	antihistamines		Dupilumab 600 mg s.c., then 300 mg q2w	Cessation of itch (NRS 0), partial resolution of skin lesions (EASI 20) at 12 wk, sustained for 12 mo follow-up	[199]
Netherton syndrome	case report (5)	1	F/20	Ichthyosis linearis circumflexa, trichorrhexis invaginata	topical steroids, antihistamines, acitretin, antibiotics		Dupilumab 600 mg s.c., then 300 mg q2w	Control of itch after 2 days, complete resolution of skin lesions (EASI 2.7) in 4 wk, improved quality of life (DLQI –20 in 6 wk). Hair regrowth after 3 mo.	[200]
Netherton syndrome	case series (4)	2	F/32, F/17	Ichthyosis linearis circumflexa (2/2), trichorrhexis invaginata (2/2), food allergies (2/2), optic nerve inflammation (1/2)	topical and systemic steroids (2/2), omalizumab (1/2)		Dupilumab 600 mg s.c., then 300 mg q2w	Reduction of itch within one day (2/2), itch NRS –4 (1/2) or –2 (1/2) sustained for 6 mo. Partial resolution of skin lesions (EASI 12–13 at 6 mo, 2/2). Hair regrowth (1/2).	[201]
Netherton syndrome	case report (5)	1	F/40	Ichthyosis linearis circumflexa, trichorrhexis invaginata, AD, asthma	topical steroids, topical retinoid, topical tacrolimus, acitretin, azathioprin, ciclosporin, IVIg		Dupilumab 600 mg s.c., then 300 mg q2w	Temporary response with improvement in pruritus and skin lesions at 6 wk. Deterioration after 8 wk, itch and skin findings worse than at baseline by wk 10.	[202]
Netherton syndrome	case series (4)	2	F/12, M/8	IVIG (1/2)		IVIG (1/2), dose reduction	Dupilumab 600 mg s.c., then 300 mg q4w and later 200 mg q2w (1/2). Dupilumab 300 mg q4w (1/2)	Reduction of itch (NRS –5 or –4 in 4 wk), sustained for 10 wk follow up. Sustained improvement of skin lesions.	[203]
Netherton syndrome	case report (5)	1	M/43	asthma, adrenal insufficiency, squamous cell carcinoma	topical and systemic steroids, MTX, MMF, azathioprin, phototherapy		Dupilumab 600 mg s.c., then 300 mg q2w	Resolution of skin manifestations (EASI –76% of baseline at wk 4), improved quality of life (DLQI 2 at wk 4)	[204]
Netherton syndrome	case report (5)	1	F/32	Ichthyosis linearis circumflexa, trichorrhexis invaginata	topical steroids, topical retinoid, topical tacrolimus, ciclosporin		Dupilumab 600 mg s.c., then 300 mg q2w	Resolution of itch (NRS –7 in 4 wk), improvement of skin lesions (BSA –50% in 4 wk)	[205]
Erythrodermic ichthyosis	case report (5)	1	M/38	AD, frequent bacterial and fungal skin infections	topical steroids, ciclosporin, MTX, acitretin	Guselkumab	Dupilumab 300 mg s.c. q2w	Improvement of ichthyosis and AD after 10 wk	[207]
Peeling skin syndrome Type 1	case report (5)	1	F/17	growth delay			Dupilumab 400 mg s.c., then 200 mg q2w	Mild reduction of erythroderma; pruritus and quality of life not affected. Reduced serum IgE	[208]
Trichothio-dystrophy (*ERCC2*-mutation)	case report (5)	1	M/8	ichthyosiform erythroderma, nail dystrophy, allergic rhinoconjuncitivitis, asthma, trichorrhexis nodosa	topical steroids and calcineurin inhibitors, antihistamines		Dupilumab 200 mg s.c. q2w	Complete remission of skin lesions, less pruritus, remission of asthma afer 12 mo	[209]
Neurofibromatosis type 1	case report (5)	1	F/30	AD	systemic steroids, ciclosporin		Dupilumab 600 mg s.c., then 300 mg q2w	Size reduction of neurofibromas at 4 wk, number and size of neurofibromas stable for 1.5 years. Remission of AD.	[210]
TTC7A-deficiency	case report (5)	1	F/5	multiple intestinal atresia, combined immunodeficiency, linear IgA-dermatosis elevated IgE and hypereosinophilia	topical and systemic steroids, antihistamines, gabapentin, clonidine, mirtazapine, amitriptyline, mepolizumab (50 mg q4w)	methylprednisolone (1 mg/kg/d max.), tapered	Dupilumab 100 mg s.c. q2w	Improvement of itch within few days, complete resolution of pruritus and skin lesions at 6 mo, sustained after withdrawal of steroid	[211]
IPEX syndrome	case report (5)	1	M/2	Vitiligo, milk protein allergy, neurofibromatosis, growth delay	bone marrow transplant, systemic steroids, sirolimus, ciclosporin, rituximab, abatacept		Dupilumab 200 mg s.c. q4w	Complete remission of skin findings at 12 wk, improved pruritus	[212]
X-linked agamma-globulinemia with AD-like eczema	case report	1	M/11	multiple episodes of skin infections	topical steroids, intravenous antibiotics, intravenous immunoglobulins		Dupilumab 300 mg s.c. q2w	Complete resolution of skin findings and pruritus, no subsequent skin infections after 3 mo	[213]

Reports of patients with various hereditary primary skin diseases including Netherton syndrome and ichthyoses were collected from databases. Evidence levels 1 through 5 were assigned to each report according to the Oxford Centre for Evidence Based Medicine and denoted in parentheses after the study type. AD, atopic dermatitis; BSA, body surface area; DLQI, dermatology life quality index; EASI, eczema area and severity index; IVIG, intravenous immunoglobulins; mo, month; MTX, methotrexate; NRS, numerical rating scale; q2w, biweekly; q4w, every 4 weeks; s.c., subcutaneous; wk, weeks.

**Table 7:** Eosinophilic dermatoses.

Disease	Study Type	n	Sex/Age	Presentation	Medical History	Eosinophilia?	Prior Therapies	Concomitant Therapy	Therapy	Response	Reference
HES	case report (5)	1	M/57	pruritic hyperpigmentated papules on head and neck. Pulmonary opacities	obesity, diabetes mellitus	Yes (1900/µL)	topical and systemic steroids, phototherapy, pegylated interferon a-2a (180 µg/wk s.c.), mepolizumab, hydroxyurea, gabapentin, antihistamines	Hydroxyurea, gabapentin, hydroxycine (doses reduced)	Dupilumab 600 mg s.c., then 300 mg q2w	Reduction of pruritus (NRS 10 to 3) and resolution of skin lesions at wk 4, improvement of pulmonary findings at 23 wk	[215]
HES	retrospective study (4)	9	42 (median, 11–85 range), females: 67%	5/9 with skin findings	AD (1/9)	Yes (>1500/µL)	Systemic steroids; Benralizumab (1/6), Omalizumab (2/9), Mepolizumab (2/9); Dupilumab was first-line biological in 4/9	Systemic steroid (5/9), tapered	Dupilumab 600 mg s.c., then 300 mg q2w	Improvement of skin lesions (3/5), no hematologic remissions (normal eosinophil count, 0/9)	[216]
HES	case report (5)	1	F/51	Generalized eczema on trunk and extremities, urticaria-like rashes, severe pruritus. Abdominal cramping pain, uncontrolled asthma.	Allergic asthma, chronic sinusitis, allergic rhinitis.	Yes (2100/µL)	Topical and systemic steroids	Systemic steroid, tapered	Dupilumab 600 mg s.c., then 300 mg q2w	Reduction of pruritus (NRS 8 to 4), increased quality of life (DLQI 25 to 9), increased FEV1	[217]
EDHM	case report (5)	1	M/82		CLL (chemotherapy)		systemic steroids			Improvement of itch (NRS 6 to 0 in 4 wk)	[8]
EDHM	case report (5)	1	M/81	erosions and pink dermal nodules on chest and face	CLL with leukemia cutis (treated with rituximab and chlorambucil)		topical and systemic steroids		Dupilumab 600 mg s.c., then 300 mg q2w	Complete resolution of skin lesions after 4 wk	[218]
EDHM	case report (5)	1	M/59	prurituc urticarial targetoid plaques on trunk and extremities with erosion and impetiginization	CLL (treated with ibrutinib)	No	topical and systemic steroid, antibiotic		Dupilumab 600 mg s.c., then 300 mg q2w	Complete clearance after 6 wk, sustained for 6 mo follow-up	[219]
EDHM	case report (5)	1	F/50s	pruritic rash on extremities with indurated papules and blisters	small lymphocytic lymphoma (treated with rituximab + bendamustine)	No	topical and systemic steroid	none	Dupilumab 600 mg s.c., then 300 mg q2w	Improvement of pruritus and complete clearance of skin lesions at wk 6	[220]
Hyper-IgE syndrome (*ZNF341* deficiency)	case report (5)	1	F/48	severe pruritus, disseminated excoriated papules and scars			topical steroids	topical steroids, discontinued	Dupilumab 600 mg s.c., then 300 mg q2w	Complete remission of skin findings (SCORAD < 10) at wk 4, improved quality of life (DLQI < 5 at wk 6)	[221]
Hyper-IgE syndrome (*STAT3*-mutation)	case report (5)	1	F/2	pruritic disseminated papules and pustules	recurrent pulmonary and skin infections	Yes (1700/µL)	topical and systemic steroids, topical tacrolimus, antibiotics, antihistamines		Dupilumab 600 mg s.c., then 300 mg q2w	Complete resolution of itch and skin lesions, sustained at 6 mo follow-up	[222]
Hyper-IgE syndrome (*STAT3*-mutation)	case report (5)	1	M/17	generalized AD-like eczema	recurrent skin and respiratory tract infections, multiple type-I allergies	Yes (>20% of leukocytes)	topical and systemic steroids, antihistamines, ciclosporin (5 mg/kg/d)	topical steroids	Dupilumab 600 mg s.c., then 300 mg q2w	Partial remission of skin findings and better quality of life (DLQI 2) after 12 mo	[223]
Hyper-IgE syndrome (*STAT3*-mutation)	case report (5)	1	M/9	generalized eczema with papules and xerosis	recurrent pneumonia, skin infections, liver abscess		topical steroids, antibiotics, antihistamines		Dupilumab 200 mg s.c., then 100 mg q2w, later q3–4w	Complete remission at wk 20 (SCORAD and EASI 0)	[224]
Hyper-IgE syndrome (*STAT3*-mutation)	case report (5)	1	F/28	Recurrent flares of eczema with severe pruritus	recurrent skin infections and pneumonia, asthma, allergic rhinoconjuncititis, multiple type-I allergies, depression, ulcerative colitis (total colectomy 4 years prior)	Yes (1910/µL)	topical and systemic steroids, topical calcineurin inhibitors, antihistamines, IVIg, ciclosporin, infliximab	IVIG	Dupilumab	Complete clearance of skin lesions	[225]
Hyper-IgE syndrome (*STAT3*-mutation)	case report (5)	1	M/21	generalized eczema	refractory diarrhea, perforated colon	Yes (6000/µL)	IVIg, systemic steroid	IVIG	Dupilumab 300 mg s.c. q3w	Complete remission of skin findings (EASI 0), cessation of diarrhea after 6 mo	[226]
Hyper-IgE syndrome (*STAT3*-mutation)	case report (5)	1	M/14	AD-like eczema	recurrent skin infections, pneumonia; eosinophilic esophagitis	Yes (800/µL)	topical steroids and calcineurin inhibitors		Dupilumab 600 mg s.c., then 300 mg q2w	Remission of skin findings (SCORAD 10) after 4 wk	[227]
Hyper-IgE syndrome (*STAT3*-mutation)	case report (5)	1	M/18	generalized eczema with lichenification	recurrent skin and respiratory tract infections		topical steroids, topical tacrolimus		Dupilumab 600 mg s.c., then 300 mg q2w, later q4w	Resolution of skin findings (EASI 60 to 9.3) and itch (NRS 9 to 2)	[228]
Hyper-IgE syndrome (*STAT3*-mutation)	case report (5)	1	F/33	generalized pruritic eczema	recurrent infections		topical steroids, topical pimecrolimus, phototherapy		Dupilumab 600 mg s.c., then 300 mg q2w	Complete remission of skin findings (SCORAD < 10) and pruritus at wk 4	[229]
Hyper-IgE syndrome (*DOCK8*-mutation)	case report (5)	1	M/13	severe generalized eczema	recurrent skin infections, asthma, allergic rhinigis, multiple type-I allergies, drug allergies		topical and systemic steroids, omalizumab, MTX, IVIG, ciclosporin	ciclosporin; IVIG (discontinued); topical and systemic steroids (discontinued)	Dupilumab 200 mg s.c. q2w	Partial sustained remission of skin findings and pruritus	[230]
Hyper-IgE syndrome (*DOCK8*-mutation)	case series (4)	2	F/11, F/10	Severe pruritic eczema on extremities and trunk	recurrent skin infections and pneumonia (2/2), type-I allergies (1/2)	Yes (>2000/µL)	topical (2/2) and systemic steroids (1/2), antihistamines, antibiotics	topical (2/2) and systemic steroids (1/2)	Dupilumab 600 mg s.c., then 300 mg q2w (1/2) or 400 mg s.c., then 200 mg q2w (1/2)	Significant improvement of pruritus and skin lesions at 4 wk, less frequent skin infections (2/2)	[231]
Kimura disease	case report (5)	1	M/57	itchy subcutaneous nodule on left arm		Yes (3520/µL)		none	Dupilumab 600 mg s.c., then 300 mg q2w, later q4w	Complete resolution at 4 wk, sustained at 10 mo	[232]
Kimura disease	case report (5)	1	M/36	subcutaneous nodule on left thigh			surgical excision		Dupilumab 600 mg s.c., then 300 mg q2w	Complete resolution, sustained at 12 mo follow-up	[233]
Kimura disease	case report (5)	1	M/59	itchy and painful subcutaneous nodules in the face	schistosomiasis (7 years prior)	Yes (15% of leukocytes)	systemic steroids, antibiotics, dapsone, indomethacin		Dupilumab 300 mg s.c. q2w	Complete resolution of skin findings at 4 wk, sustained for 6 mo follow-up	[234]
Kimura disease	case report (5)	1	M/57	bilateral auricular subcutaneous masses		Yes (1640/µL, 22.8% of leukocytes)	systemic steroid, omalizumab		Dupilumab 600 mg s.c., then 300 mg q2w	Size reduction at 16 wk follow-up	[235]
Papulo-erythro-derma Ofuji	case series (4)	2	M/80s, M/90s	pruritic confluent papules, erythroderma, deck-chair sign		Yes	topical and systemic steroids		Dupilumab 600 mg s.c., then 300 mg q2w, later q4w (1/2) or q6w (1/2)	Complete resolution of skin lesions and pruritus (NRS 10 to 0) at 16 wk (2/2)	[236]
Papulo-erythro-derma Ofuji	case report (5)	1	M/65	disseminated pruritic papules	no AD or atopy	Yes (1000/µL)	topical and systemic steroids		Dupilumab 600 mg s.c., then 300 mg q2w	Complete resolution of skin lesions and pruritus (NRS 0) at 14 wk	[237]
Papulo-erythro-derma Ofuji	case report (5)	1	M/80s	disseminated pruritic papules		Yes (24.8% of leukocytes)	antihistamines, topical steroids, minocycline, antibiotics, ciclosporin		Dupilumab	Complete resolution of skin lesions and pruritus at 6 mo	[238]
Wells syndrome	case report (5)	1	F/52	pruritic morphea-like indurated plaques	eosinophilic asthma, nasal polyps		dapsone, systemic steroids, benralizumab	systemic steroids, tapered	Dupilumab	Complete resolution after 6 mo, improvement of asthma	[239]
Wells syndrome	case report (5)	1	F/80	relapsing painful arcuate patches and plaques			systemic steroids, dapsone, hydroxychloroquine, doxycycline, ciclosporin	dapsone 100 mg/d	Dupilumab 400 mg s.c., then 200 mg q2w	Resolution of skin lesions after 5 wk	[240]
EAE	case report (5)	1	F/14	annular urticarial plaques on trunk, arms and forehead with severe pruritus with central hyperpigmentation	none	Yes (700/µL, 11.3% of leukocytes)	topical and systemic steroids, dapsone, tofacitinib		Dupilumab 600 mg s.c., then 300 mg q2w	Complete resolution of skin lesions and pruritus after 4 wk	[241]
EAE	case report (5)	1	F/56	annular plaques on trunk and arms with polycyclic margins and central hyperpigmentation, intense pruritus	none	No	topical and systemic steroids, ciclosporin, hydroxychloroquine, dapsone, MTX, thalidomide, indomethacin	systemic steroid, tapered	Dupilumab 600 mg s.c., then 300 mg q2w	Complete remission after 4 wk	[242]
Eosino-philic fasciitis	case report (5)	1	M/46	Swelling and induration of skin on abdomen and arms, groove sign of superficial veins		Yes (1110/µL)	Systemic steroid	Systemic steroid, tapered	Dupilumab 300 mg s.c. q2w	Resolution of skin findings (clinically and in MRI) after 10 wk	[243]
Angiolym-phoid hyperplasia with eosino-philia	case report (5)	1	F/68	diffuse pruritic dermal nodules on face, arms and trunk		Yes (1200/µL)	Mepolizumab, benralizumab		Dupilumab 300 mg s.c. q2w	Resolution of skin findings after 4 wk. Dupilumab discontinued after 6 mo with sustained response.	[244]

Reports of patients with various types of dermatoses characterized by peripheral eosinophilia were collected from databases. Evidence levels 1 through 5 were assigned to each report according to the Oxford Centre for Evidence Based Medicine and denoted in parentheses after the study type. AD, atopic dermatitis; CLL, chronic lymphatic leukemia; DLQI, dermatology life quality index; EAE, erythema annulare eosinophilicum; EASI, eczema area and severity index; EDHM, eosinophilic dermatosis of hematologic malignancy; FEV1, forced exspiratory volume in 1 s; HES, hypereosinophilia syndrome; IVIG, intravenous immunoglobulins; mo, month; MRI, magnetic resonance imaging; MTX, methotrexate; NRS, numerical rating scale; q2w, biweekly; q4w, every 4 weeks; s.c., subcutaneous; SCORAD, SCORing atopic dermatitis index; wk, weeks.

**Table 8:** Connective tissue disorders and other inflammatory skin diseases.

Disease	Study Type	n	Sex/Age	Medical History	Prior Therapies	Concomitant Therapy	Therapy	Response	Reference
Lichen sclerosus	case report (5)	1	F/80		topical steroids, MMF (2 g/d), phototherapy, MTX (15 mg/wk)		Dupilumab 600 mg s.c., then 300 mg q2w	Reduction of itch and skin lesions after 12 wk, complete resolution after 10 mo	[245]
Dermatomyositis	case report (5)	1	F/28		topical and systemic steroids, dapsone, MTX, azathioprin, hydroxychloroquine, thalidomide		Dupilumab 600 mg s.c., then 300 mg q2w	No improvement of pruritus, discontinued	[246]
IgG4-related disease	case report (5)	1	M/67	AD, allergic rhinoconjunctivitis, asthma, obstructive sleep apnea	systemic steroids		Dupilumab 600 mg s.c., then 300 mg q2w	Resolution of retroperitoneal fibrosis and skin lesions after 12 mo	[247]
Keloid	case report (5)	1	M/53	Severe AD	intralesional steroids		Dupilumab 300 mg s.c. q2w	Drastic size reduction	[248]
Keloid	case report (5)	1	F/37				Dupilumab 600 mg s.c., then 300 mg q2w	Reduction of pain, no size reduction of keloid	[249]
Keloid	case series (4)	8	32.5 (median, 23–52 range), females: 2/8				Dupilumab 300 mg s.c. q2w	No effects or deterioration	[250]
Keloid	case series (4)	2	F/17, M/17	acne vulgaris, folliculitis	intralesional steroids (2/2), isotretinoin (1/2), doxycycline (1/2)		Dupilumab 300 mg s.c. q2w	No improvement of pain, itch or size of keloid	[251]
CAD	case series (4)	4	42.6 (median, 25–59 range), females: 2/4		topical (4/4) or systemic (2/4) steroids, topical calcineurin inhibitors (1/4) antihistamines (1/4), azathioprine (2/4), ciclosporine (3/4), hydroxychloroquine (3/4), MTX (2/4), thalidomide (1/4), MMF (1/4)	photoprotection (4/4), topical steroids (2/4), azathioprine (1/4, tapered), hydroxychloroquine (1/4), MTX (1/4, tapered), ciclosporin (1/4, tapered), thalidomide (1/4)	Dupilumab 600 mg s.c., then 300 mg q2w	Reduced itch and skin lesions by week 2–8 (4/4)	[252]
CAD	case series (4)	5	66 (median, 49–79 range), females: 1/5		topical (5/5) or systemic (2/5) steroids, hydroxychloroquine (1/5), MMF (5/5), MTX (1/5), azathioprine (1/5)	none	Dupilumab 600 mg s.c., then 300 mg q2w	Significant improvement of itch and skin lesions (5/5); dupilumab discontinued due to facial erythema (1/5)	[253]
CAD	case report (5)	1	M/45	AD	topical and systemic steroids, MTX, antihistamines, ciclosporine (75 mg/d), hydroxychloroquine (0.2 g/d)	ciclosporine (75 mg/d, tapered)	Dupilumab 600 mg s.c., then 300 mg q2w	Resolution of pruritus at 8 wk, reduction in skin lesions, improved quality of life (DLQI 17 to 2)	[254]
CAD	case report (5)	1	M/82		topical steroids, antihistamines, hydroxychloroquine	topical steroids, hydroxychloroquine (400 mg/d)	Dupilumab 600 mg s.c., then 300 mg q2w	Clearance of skin lesions at 16 wk	[255]
CAD	case report (5)	1	M/60	no atopy	topical and systemic steroids, MTX (25 mg/wk), ciclosporin (5 mg/kg/d), azathioprine (100 mg/d)	MTX (15 mg/wk)	Dupilumab 600 mg s.c., then 300 mg q2w	Complete remission after 5 mo	[256]
CAD	case series (4)	3	M/58, M/77, M/69	AD (1/3)	topical (3/3) and systemic (2/3) steroid, MTX (2/3), antihistamines (2/3), hydroxychloroquine (3/3), azathioprine (1/3), MMF (1/3)	hydroxychloro-quine 200 mg/d (3/3)	Dupilumab 600 mg s.c., then 300 mg q2w	Complete resolution (3/3)	[257]
CAD	case report (5)	1	M/54	AD, allergic rhinitis, alopecia universalis	topical and systemic steroids, MTX (15 mg/wk), MMF (1 g/d), azathioprine (50 mg/d), hydroxychloroquine (400 mg/d), apremilast (60 mg/d), ciclosporine (200 mg/d)	pulsed prednisone, topical steroids	Dupilumab 600 mg s.c., then 300 mg q2w	Partial response at 9 mo	[258]
Actinic prurigo	case report (5)	1	F/7	cheilitis	antihistamines, topical steroids, ciclosporin (5 mg/kg/d), MTX (0.4 mg/kg/wk)	MTX, discontinued after 8 wk	Dupilumab 400 mg s.c., then 200 mg q2w	50% improvement in pruritus at 4 wk, resolution of skin lesions at 8 wk	[259]
GVHD	case series (4)	4	6.5 (median, 4–17 range), females: 3/4	cord blood transplantation (3/4), peripheral stem cell transplantation (1/4)	topical (4/4) and systemic (1/4) steroids, topical calcineurin inhibitors (3/4), phototherapy (1/4), systemic tacrolimus (3/4), MMF 3/4), ruxolitinib (2/4)		Dupilumab 400 mg s.c., then 200 mg q4w (2/4), 200 mg s.c. q2w (1/4), 600 mg s.c., then 300 mg q3w (1/2)	Complete resolution (IGA 0; 3/4), no response (1/4)	[260]
Palmoplantar pustulosis	case report (5)	1	M/52	non-atopic	topical steroids, topical vitamin D-derivatives, secukinumab (300 mg qw)		Dupilumab 300 mg s.c. q2w	Complete resolution after 4 wk	[261]
Lichen planus	case report (5)	1	M/52	no atopy	topical and systemic steroids, acitretin		Dupilumab 600 mg s.c., then 300 mg q2w	Improvement of pruritus (NRS 9 to 1), improvement of skin lesions	[8]
Lichen planus	case report (5)	1	F/92		topical and systemic steroids		Dupilumab 600 mg s.c., then 300 mg q2w	Ccomplete clearance of skin lesions and pruritus after 4 wk	[262]
Lichen planus	case report (5)	1	M/52	AD	topical and systemic steroids, acitretin		Dupilumab 600 mg s.c., then 300 mg q2w	Partial resolution of skin lesions, resolution of itch (NRS 1/10) after 12 wk	[263]
HS	case report (5)	1	M/25	severe AD	ciclosporin (200 mg/d), minocycline (100 mg/d)	systemic clindamycin	Dupilumab 600 mg s.c., then 300 mg q2w	IHS4 < 3 after 12 mo	[264]
HS	case report (5)	1	M/43	AD	ciclosporin, lymecycline		Dupilumab 600 mg s.c., then 300 mg q2w	No flare of HS in 6 mo	[265]
HS	case report (5)	1	M/50	AD	adalimumab		Dupilumab 600 mg s.c., then 300 mg q2w	HiSCR achieved after 16 wk	[266]
Granuloma anulare	case report (5)	1	F/74		topical steroids, hydroxychloroquine, MTX, niacinamide, adalimumab, antibiotics		Dupilumab 600 mg s.c., then 300 mg q2w	Nearly complete clearance of skin lesions after 4 wk	[267]
Lichen amyloido-sus	case report (5)	1	F/49	AD	topical steroids, antihistamines		Dupilumab	Flattening of LA papules and reduction of itch	[268]
Lichen amyloido-sus	case series (4)	2	F/28, F/30	AD	topical steroids, topical calcineurin inhibitors, phototherapy, ciclosporin (all: 1/2)		Dupilumab	Complete resolution of skin lesions and pruritus (NRS 2) after 5 or 6 mo	[269]
Lichen amyloido-sus	case report (5)	1	M/76	no atopy	phototherapy, amitriptyline, antihistamines, acitretin, benralizumab		Dupilumab 600 mg s.c., then 300 mg q2w	Flattening of papules, resolution of pruritus after 12 wk	[270]
Food allergy	case report (5)	1	F/30	AD, allergic rhinitis, anaphylactic shock after ingestion of corn, several food type-I sensitivities			Dupilumab 600 mg s.c., then 300 mg q2w	Oral provocation with peanuts and corn with no reaction after 3 mo	[271]
Peanut allergy	interventional study (3)	24	11.7 (mean, 3.28 SD), females: 6/24				Dupilumab	Double-blind placebo-controlled food challenge (DBPCFC) passed by 8.3%	NCT 03793608

Records of patients with inflammatory connective tissue disorders or other inflammatory skin diseases not classified before were acquired from clinicaltrials.gov and databases. Evidence levels 1 through 5 were assigned to each report according to the Oxford Centre for Evidence Based Medicine and denoted in parentheses after the study type. AD, atopic dermatitis; CAD, chronic actinic dermatosis; DLQI, dermatology life quality index; GVHD, graft versus host disease; HiSCR, hidradenitis suppurativa clinical response; HS, hidradenitis suppurativa; IGA, investigator global assessment; IHS4, International Hidradenitis Suppurativa Severity Score System; LA, lichen amyloidosis; MMF, mycophenolate mofetil; mo, month; MTX, methotrexate; NRS, numerical rating scale; q2w, biweekly; q3w, every 3 weeks; q4w, every 4 weeks; s.c., subcutaneous; wk, weeks.

**Table 9:** Cutaneous lymphoma.

Disease	Study Type	n	Sex/Age	Medical History	Prior Therapies	Concomitant Therapy	Therapy	Response	Reference
CTCL (Sézary), St. IVA1 (T4, N0, M0, B2)	case report (5)	1	M/68	AD	phototherapy, ECP, bexarotene, interferon a-2b, topical steroids	phototherapy, ECP, bexarotene, interferon a-2b, topical steroids	Dupilumab 600 mg s.c., then 300 mg q2w	Reduction of blood involvement, partial resolution of skin findings, improvement of itch after 12 wk	[272]
CTCL (Sézary), St. IVA1 (pT4, N1, B2, M0)	case report (5)	1	F/74		phototherapy, topical and systemic steroids, ciclosporin, ECP, interferon a-2a	ECP	Dupilumab 600 mg s.c., then 300 mg q2w	Improvement of pruritus (NRS 2) and quality of life (DLQI) within days	[273]
CTCL (MF) (1/2), Sézary (1/2)	case series (4)	2	F/37, M/55	AD (1/2)	topical steroids, phototherapy, ciclosporin (1/2)		Dupilumab	MF (1/2): improvement of pruritus and partial remission of MF after 16 wk, Sézary (1/2): no effect	[274]
CTCL (MF) St. IIB (T3 N0 M0 B0)	case report (5)	1	F/51	no AD	azathioprin, topical and systemic steroids		Dupilumab	Relief of pruritus but spreading of plaques and new skin tumors.	[275]
CTCL (MF) St. IB-IIIB	case series (4)	7	65.6 (median, 40–77 range), females: 3/7				Dupilumab	Initial improvement (median duration 2 mo, 6/7). Subsequent progression (7/7), progression into Sézary syndrome (3/7), death (2/7)	[276]
CTCL (Sézary), St. IVA1 (T4, N2, M0, B2)	case report (5)	1	F/48	AD			Dupilumab	CTCL developed under therapy with dupilumab. Therapy was discontinued.	[277]
CTCL (MF) St. ≥ III (T4 Nx M0 Bx)	case report (5)	1	F/47	AD				CTCL developed under therapy with dupilumab. Therapy was discontinued.	[278]
CTCL (MF) St. IB (T2 N0 M0 B0)	case report (5)	1	M/58	AD, allergic rhinitis, conjunctivitis, asthma	topical steroids		Dupilumab 600 mg s.c., then 300 mg q2w	Exacerbation under dupilumab	[279]
CTCL (MF) St. IIA (T2 N1 M0 B0)	case report (5)	1	M/72		topical steroids and calcineurin inhibitors			Progression under dupilumab	[280]
CTCL (MF) St. IB (2/2)	case series (4)	2	F/48, M/55	AD (2/2)	topical steroids (2/2), MTX (1/2), phototherapy (2/2)		Dupilumab	No effect, discontinued after 5 or 6 mo	[281]
CTCL (Sézary), St. IVA1 (T4 N0 M0 B2)	case report (5)	1	M/64	AD	topical steroids, phototherapy		Dupilumab 600 mg s.c. single dose	CTCL developed under dupilumab for AD	[282]
Cutaneous B cell pseudolymphoma	case report (5)	1	M/76		topical steroids, rituximab, hydroxychloroquine, MTX		Dupilumab 600 mg s.c., then 300 mg q2w	Complete resolution of skin findings at 6 wk	[283]
CTCL (MF) St. IB (T2b N0 M0 B0), lichenoid drug eruption associated with mogamulizumab	case report (5)	1	F/26		topical and systemic steroids, antihistamines	Mogamulizumab discontinued	Dupilumab 600 mg s.c., then 300 mg q2w	Complete resolution of drug eruption and improvement of itch after 4 mo	[284]

Collated reports of patients with cutaneous lymphoma treated with dupilumab. Evidence levels 1 through 5 were assigned to each report according to the Oxford Centre for Evidence Based Medicine and denoted in parentheses after the study type. AD, atopic dermatitis; CTCL, cutaneous T cell lymphoma; ECP, extracorporeal photopheresis; MF, mycosis fungoides; mo, month; MTX, methotrexate; q2w, biweekly; s.c., subcutaneous; St, stage; wk, weeks.

## Data Availability

No new data were created or analyzed in this study. Data sharing is not applicable to this article.
